# Leaf isoprene and monoterpene emissions vary with fast-slow carbon economics strategies in central Amazon woody species

**DOI:** 10.3389/fpls.2025.1561316

**Published:** 2025-05-27

**Authors:** Michelle Robin, Eliane Gomes Alves, Tyeen C. Taylor, Débora Pinheiro Oliveira, Sérgio Duvoisin, José Francisco C. Gonçalves, Jochen Schöngart, Florian Wittmann, Maria T. F. Piedade, Susan Trumbore, Juliana Schietti

**Affiliations:** ^1^ Department of Biogeochemical Processes, Max Planck Institute for Biogeochemistry, Jena, Germany; ^2^ Post-Graduation Program in Ecology, National Institute of Amazonian Research, Manaus, Brazil; ^3^ Post-Graduation Program in Climate and Environment, National Institute of Amazonian Research, Manaus, Brazil; ^4^ Department of Civil and Environmental Engineering, University of Michigan, Ann Arbor, MI, United States; ^5^ Biology Department, University of Miami, Coral Gables, FL, United States; ^6^ Department of Chemistry, University of the State of Amazonas, Manaus, Brazil; ^7^ Coordination of Environmental Dynamics, National Institute of Amazonian Research, Manaus, Brazil; ^8^ Department of Wetland Ecology, Karlsruhe Institute of Technology, Rastatt, Germany; ^9^ Department of Biology, Federal University of Amazonas, Manaus, Brazil

**Keywords:** volatile isoprenoids, plant functional traits, plant functional strategies, Amazon forest, BVOCs

## Abstract

Plant responses to stress, inter-organismal signaling, and atmospheric chemistry are significantly influenced by leaf volatile isoprenoid (VI) emissions (e.g., isoprene and monoterpenes). Despite their critical roles in ecology and the atmosphere, we have little understanding of whether and how VI emissions vary with axes of plant functional variation. Understanding these relationships is particularly important in tropical forests, which emit more VIs into the atmosphere than any other biome, and where high species diversity necessitates the imputation of plant traits based on functional and evolutionary relationships. Here, we investigated how VI emissions varied with functional trait axes of fast-slow carbon economics strategies (CES) in Central Amazon Forest woody species. We measured leaf-level isoprene and monoterpene emission capacity (*E*_c_; emission measured under standard conditions of photosynthetically active radiation of 1000 µmol m^-2^ s^-1^ and leaf temperature of 30 ˚C), and 12 leaf and four stem functional traits for 91 trees from 31 species of angiosperm distributed across different vegetation types: non-flooded upland, white sand, and ancient non-flooded river terrace forests. Principal component analysis (PCA) of functional traits revealed two partially independent main axes of CES: a first axis of leaf strategies and a second of mixed leaf/stem strategies. The capacity to emit monoterpenes was observed in 27 species, and monoterpene emitters occupied the whole range of fast-slow strategies, but magnitudes of monoterpene *E*_c_ increased toward faster leaves. The capacity to emit isoprene was observed in 14 species, and isoprene emitters tended to be positioned toward slower leaf/stem strategies, with magnitudes of isoprene *E*_c_ also increasing toward slower leaves/stems. Our results highlight the importance of understanding leaf-level emissions to accurately estimate VI fluxes and provide a holistic view of emissions within CES on different organ-system levels. This shows a direction for improving current modeling estimates, which have simplified plant functional type representations and are poorly developed for compounds other than isoprene in the tropics. A more mechanistic representation of plant functional types based on forest functional compositions can reduce modeling emission uncertainties and contribute to understanding the roles of VIs within forest-atmosphere interactions, atmospheric chemistry, and the carbon cycle.

## 1 Introduction

Isoprenoids are the most abundant and chemically diverse group of secondary plant metabolites with important roles in primary and secondary metabolism (Thulasiram et al., 2007). They regulate plant physiological functions, protect against biotic and abiotic stress, take part in plant-plant signaling, and interfere in plant-herbivore interactions (Singsaas et al., 1997; Pichersky and Gershenzon, 2002; Gershenzon and Dudareva, 2007; Laothawornkitkul et al., 2008; Fineschi and Loreto, 2012; Xiao et al., 2012; Pollastri et al., 2014, 2019; Monson et al., 2021; Dani et al., 2022). Isoprene and monoterpenes are highly emitted volatile isoprenoids (VIs), accounting for the largest share of global biogenic volatile organic compound (BVOC) emissions (Guenther et al., 2006, 2012). Upon entering the atmosphere, these compounds have major influence over atmospheric chemical and physical processes, including secondary organic aerosol formation, which can affect the radiative balance of the Earth (Pöschl et al., 2010; Kulmala et al., 2013).

Tropical forests contribute to around 80% of global BVOC fluxes (Guenther et al., 2012), and the Amazon Forest, with its high plant biomass and species diversity (Cardoso et al., 2017), is considered the greatest and most diverse source of emissions. However, estimating VI fluxes from this forest is challenging due to an insufficient understanding of physiological and ecological controls on forest emissions, and the forest’s huge diversity of plant species and functional strategies (Alves et al., 2016, 2018; Taylor et al., 2018, 2021). Even though the existing knowledge on the ecophysiology of VI emissions has considerably increased over the last two decades (Morfopoulos et al., 2013; Sharkey and Monson, 2017; Alves et al., 2018; Taylor et al., 2018, 2021; Yáñez-Serrano et al., 2020; Monson et al., 2021; Gomes Alves et al., 2022, 2023; Robin et al., 2024), we still do not know exactly how isoprene and monoterpene emissions vary with plant functional strategies (Sardans et al., 2010; Harrison et al., 2013; Dani et al., 2014; Llusia et al., 2014; Fernández-Martínez et al., 2018), particularly in Amazonian woody species.

Isoprene (C_5_H_8_) emissions are light and temperature-dependent, and primarily produced from recently assimilated photosynthetic carbon (*de novo* synthesis) (Sharkey and Monson, 2017). Isoprene is mostly known for its role in thermoprotection (Singsaas et al., 1997; Pollastri et al., 2014, 2019), which has been attributed to increased thylakoid membrane stability (Velikova et al., 2011; Harvey et al., 2015) or direct/indirect antioxidant properties (Morfopoulos et al., 2013, 2014; Rodrigues et al., 2020; Velikova, 2008). More recently, multi-omic studies have demonstrated that the capacity to emit isoprene is associated with changes in gene expression and transcription factors that participate in the production of various growth and defense-related compounds (Behnke et al., 2010; Harvey and Sharkey, 2016; Lantz et al., 2019; Zuo et al., 2019; Frank et al., 2021; Monson et al., 2021; Dani et al., 2022; Srikanth et al., 2024; Weraduwage et al., 2024). These studies propose that the capacity to emit isoprene is a trait that mediates photosynthetic resource supply and metabolite demands in the face of climate stress (Monson et al., 2021).

Monoterpenes (C_10_H_16_) have been more frequently associated with slow, constitutive emissions from storage pools formed inside specialized structures, and with stronger stress-induced emissions upon breakage of these pools (e.g., insect attacks) (Arneth and Niinemets, 2010; Niinemets et al., 2013; Rasulov et al., 2019; Nagalingam et al., 2023). Monoterpene emissions exert diverse chemical signaling roles in direct and indirect defense against herbivores and plant communication (Gershenzon and Dudareva, 2007; Fineschi and Loreto, 2012; Xiao et al., 2012). However, many studies have reported leaf-level light-dependent *de novo* monoterpene emissions, especially in tropical forests, and a role in oxidative stress protection similar to isoprene has been proposed for these compounds (Kuhn et al., 2004; Jardine et al., 2015, 2017, 2020; Gomes Alves et al., 2022; Bourtsoukidis et al., 2024). While VIs are not essential for plant growth and survival (Owen and Peñuelas, 2005), the capacity to produce these compounds can be a major advantage for plant survival since they confer protection against many biotic and abiotic stressors (Loreto and Schnitzler, 2010; Fineschi and Loreto, 2012; Monson et al., 2021; Srikanth et al., 2024). Yet, VI production demands resources (carbon) that could otherwise be allocated toward plant respiration and growth (Delwiche and Sharkey, 1993; Affek and Yakir, 2003; Loreto et al., 2004; Huang et al., 2017; Lehmanski et al., 2024). Similarly, higher production of *de novo*-emitted compounds would be generally associated with a greater reallocation of photosynthetic carbon to VI production (Delwiche and Sharkey, 1993; Loreto et al., 1996).

Isoprene and light-dependent monoterpenes are both produced in the chloroplastic methyl-erythritol 4-phosphate (MEP) pathway, having dimethylallyl pyrophosphate (DMAPP) as a common precursor (Zhao et al., 2013). Monoterpenes not only contain double the amount of carbon atoms per molecule compared to isoprene, but also present a higher complexity in chemical structure (e.g., acyclic, monocyclic, bicyclic) (Thulasiram et al., 2007), and are produced one chemical reaction step later than that of isoprene (Zhao et al., 2013). This suggests that monoterpenes incur higher construction costs on both mass and energy bases. However, the affinity of isoprene synthase for DMAPP is much lower (Michaelis-Menten coefficient, *K*_m_ = 0.3 - 2.45 mM) than that of geranyl pyrophosphate synthase (GPS) for DMAPP (*K*_m_ = 0.014 - 0.037 mM) and that of monoterpene synthases for geranyl pyrophosphate (GPP) synthesized by GPS (*K*_m_ = 0.006 - 0.009 mM) (Harrison et al., 2013). Hence, even though isoprene is produced earlier in the MEP pathway, DMAPP is more easily converted to monoterpenes than to isoprene (Harrison et al., 2013). This means that a higher affinity of monoterpene synthases for DMAPP might counteract a possible lower carbon cost bias in favor of isoprene production.

Still, VIs share the same biochemical pathway of essential isoprenoids like plant hormones (abscisic acid and gibberellins) and photosynthetic pigments (chlorophyll and carotenoids) (Laule et al., 2003), and it has been suggested that VI emissions are “opportunistic” in the sense that they make use of the available DMAPP surplus when the demands for essential isoprenoid production are met (Owen and Peñuelas, 2005). However, some studies have also demonstrated that, under abiotic stress, the amount of photosynthetic carbon loss with isoprene and light-dependent monoterpene emissions can account for up to 20 and 7.4% of photosynthesis, respectively (Bamberger et al., 2017; Robin et al., in prep.). Such high percentages of photosynthetic carbon loss in stress conditions suggest that plants can also actively allocate carbon toward VI emissions despite metabolite demands downstream of the MEP pathway, which emphasizes the physiological importance of these compounds.

Therefore, a balance between the benefits for plant defense - and survival - and the associated carbon costs of isoprene and light-dependent monoterpene production possibly illustrates an ecological tradeoff that may allow evaluating their emissions from a functional ecology perspective. Functional ecology classifies and identifies organisms by sets of measurable characteristics (i.e., functional traits) that impact individual performance (e.g., growth, development, defense, survival, and reproduction) (Violle et al., 2007). Resource allocation strategies describe how plants balance growth and differentiation to optimize fitness through correlations in traits related to resource acquisition, processing, and conservation. These strategies ultimately inform the conceptual framework of carbon economics strategies (CES) (Reich, 2014), which explains how plants allocate carbon to various functions to maximize growth or survival. Within CES, the leaf economics spectrum captures tradeoffs in leaf traits related to carbon acquisition and conservation, classifying species from fast-growing, acquisitive: ones with lower leaf mass per area (LMA), higher nitrogen and phosphorus per dry mass (*N*_mass_ and *P*_mass_), and higher photosynthetic capacities (*A*_max_) - traits that enable faster returns (i.e. higher growth rates) upon assimilated carbon; to slow-growing, conservative species: with higher LMA, lower *N*_mass_ and *P*_mass,_ and lower *A*_max_ - providing slower returns but greater tissue longevity (i.e. lower mortality rates) (Wright et al., 2004). Similarly, the wood economics spectrum encompasses variation in stem traits, where slow-growing species show dense, durable wood that supports long-term survival; in contrast with fast-growing species that show lighter, less dense wood that favors rapid growth (Chave et al., 2009).

Harrison et al. (2013) showed that isoprene emission rates increase with specific leaf area (SLA), short leaf lifespan, and *A*_max_, and decrease with increased constitutive monoterpene emissions. This led to an association between isoprene and faster-growth strategies, and the hypothesis that monoterpene emissions would be associated with slower-growth strategies due to their comparably higher production costs and longer-term protective role (as these are storable compounds) against herbivores - although isoprene has been shown as involved in herbivore deterrence as well (Visakorpi et al., 2018; Pollastri et al., 2021). Likewise, Dani et al. (2014) proposed that deciduous trees (i.e., faster leaves) would be high isoprene emitters, but that evergreen trees (i.e., slower leaves) would be high monoterpene emitters - though this construct may be biased by the rarity of evergreen angiosperms in the temperate zone. Although these studies have attempted to position changes in VI emissions within CES, they are generally biased toward temperate forest species due to the higher availability of data for this biome. Moreover, studies focusing on tropical woody species are few and unclear (Sardans et al., 2010; Llusia et al., 2014) and relationships between the capacity to emit and the magnitude of VI emissions and CES in Central Amazon Forest woody species have not yet been evaluated.

Here we investigated how leaf-level VI emission capacity (*E*_c_ = emission measured at standard conditions of photosynthetically active radiation of 1000 µmol m^-2^ s^-1^ and leaf temperature of 30 ˚C) varies with fast-slow CES strategy axes derived from correlations between 16 functional traits (12 leaf traits and four stem traits) measured *in situ* for 91 trees from 31 species of angiosperm in a central Amazon site. Considering the positive impacts of VI emissions on plant defense and survival, and their associated carbon production costs, we hypothesized that (H1) both the capacity to emit and the magnitude of VI *E*_c_ vary with CES and are either (H1a) associated with faster strategies due to higher photosynthetic rates required to cover carbon demands of emissions or (H1b) associated with slower strategies where higher carbon demands from emissions are compensated by greater leaf longevity; and that (H2) both the capacity to emit and the magnitude of isoprene and monoterpene *E*_c_ vary simultaneously with CES in the sense that either (H2a) the capacity to emit isoprene and monoterpenes or the magnitude of their *E*_c_ are mutually exclusive, as the increasing carbon cost of one must be compensated by a decreasing cost of the other, or (H2b) the capacity to emit isoprene and monoterpenes or the magnitude of their *E*_c_ are positively associated due to their complementary roles in oxidative stress protection.

## 2 Materials and methods

### 2.1 Study site

This study was conducted at the Amazon Tall Tower Observatory (ATTO) and PELD-MAUA (PELD is the acronym in Portuguese for Long Term Ecological Research Project) experimental plots. Plots are located in the central Amazon region, at the Uatumã Sustainable Development Reserve (USDR), about 150 km northeast of the city of Manaus (S 02 08.9° W 059 00.2°) (**Supplementary Figure S1**). The climate is tropical humid, with a mean annual temperature of 26.7 °C and precipitation of 2376 mm, being characterized by a pronounced rainy season from November-May and a drier season from June-October (Andreae et al., 2015). The reserve covers 424,430 ha with a mosaic of non-flooded upland (locally called *terra firme*), white sands, and ancient non-flooded river terrace forests (Yáñez-Serrano et al., 2015).

### 2.2 Sampling design

We measured leaf and stem functional traits and VI *E*_c_ for 91 trees from 31 previously identified (with individual vouchers collected) and confirmed taxonomic woody species belonging to 16 families of angiosperms. Species were chosen from a preliminary selection based on their presence and abundance (in number of individuals per species) in the PELD-MAUA woody species inventory (Andreae et al., 2015) and available functional trait data for wood density and potential growth rate, obtained from local, regional, and global databases - Zanne et al. (2009); Reserva Adolpho Ducke Permanent Plots (PPBio, 2012); TRY Database (Kattge et al., 2011). Despite this preliminary selection, we measured functional traits *in situ* rather than relying on existing trait databases to obtain more realistic and comprehensive functional strategies for woody species prevalent in the Central Amazon Forest, many of which lacked data in current databases.

Species were distributed across four permanent plots along the ATTO access road (**Supplementary Figure S1**) covering: non-flooded upland (locally called *terra firme*, one plot); white sand (two plots); and ancient non-flooded river terrace (one plot) forests. These vegetation types are characterized by differences in soil and vegetation attributes. *Terra firme* vegetation is dense and mature, with a mean canopy height of 35 m (Gomes Alves et al., 2023), situated over highly weathered and well-drained ferralsols (Chauvel et al., 1987). White sand vegetation covers shrubby and forested physiognomies, with canopies that reach from 12 to 20 m in height and various degrees of stratification (Demarchi et al., 2022). This vegetation sits on top of arenosols characterized by high water permeability, low water holding capacity, low specific heat capacity, and often a nutrient-poor organic layer (Quesada et al., 2011). River terrace soils are alisols, with a more recent pedogenetic status than *terra firme* ferralsols and a greater capacity to supply nutrients (Andreae et al., 2015).

We sampled species in plots of different vegetation types to maximize variation among species and allow capturing a broader range of CES. Since most species did not occur in all vegetation types, we could not repeat species across different vegetation types. In the case where a species would occur in more than one vegetation type, we sampled the species in the vegetation type where it was the most representative in terms of number of individuals and basal area. We sampled 2–5 trees for each species and selected trees that showed similar DBH values and represented maximum DBH values for their species at the plot (**Table 1**). Some species had fewer replicates than others due to technical issues in obtaining measurements of VI *E*_c_ and stomata-related traits for some individuals.

**Table 1:** Taxonomic species, family, vegetation type (*Terra firme*, dense non-flooded upland forest; White sand, white sand forest; River terrace, ancient non-flooded river terrace forest), range of diameter (minimum-maximum) at 1.3 m height (DBH; cm), and the number of trees measured per species for 31 Amazonian woody species evaluated in this study.

Species	Family	Vegetation type	DBH (min-max)	Trees/Species
*Bocageopsis pleiosperma*	Annonaceae	River terrace	16.2 - 28.7	3
*Aspidosperma carapanauba*	Apocynaceae	*Terra firme*	42.7 - 51.7	2
*Geissospermum sericeum*	Apocynaceae	*Terra firme*	17.2 - 51.0	3
*Macoubea* sp*rucei*	Apocynaceae	White-sand	14.2 - 21.3	3
*Protium grandifolium*	Burseraceae	*Terra firme*	13.9 - 19.0	4
*Protium hebetatum*	Burseraceae	White sand	13.5 - 22.2	5
*Pourouma minor*	Cecropiaceae	River terrace	12.0 – 17.0	2
*Croton matourensis*	Euphorbiaceae	River terrace	17.3 - 22.0	3
*Aldina heterophylla*	Fabaceae	White sand	29.4 - 51.5	3
*Inga alba*	Fabaceae	River terrace	38.5 - 60.5	2
*Macrolobium duckeanum*	Fabaceae	White sand	10.4 - 12.8	3
*Parkia igneiflora*	Fabaceae	White sand	12.3 - 19.0	3
*Swartzia reticulata*	Fabaceae	*Terra firme*	37.4 - 38.8	2
*Corythophora rimosa*	Lecythidaceae	*Terra firme*	32.3 - 40.5	3
*Eschweilera coriacea*	Lecythidaceae	White sand	12.8 - 21.4	5
*Eschweilera grandiflora*	Lecythidaceae	*Terra firme*	11.0 - 39.3	5
*Pachira faroensis*	Malvaceae	White sand	12.7 - 17.2	3
*Scleronema micranthum*	Malvaceae	*Terra firme*	15.0 - 20.2	3
*Theobroma sylvestre*	Malvaceae	River terrace	10.1 - 12.0	3
*Mouriri duckeana*	Melastomataceae	River terrace	11.5 - 47.0	2
*Trichilia schomburgkiana*	Meliaceae	River terrace	16.0 - 17.3	2
*Naucleopsis caloneura*	Moraceae	*Terra firme*	14.5 - 17.0	3
*Minquartia guianensis*	Olacaceae	River terrace	34.5 - 41.0	3
*Pagamea coriacea*	Rubiaceae	White sand	14.0 - 16.1	3
*Chrysophyllum sanguinolentum*	Sapotaceae	White sand	22.0 - 35.7	3
*Ecclinusa guianensis*	Sapotaceae	*Terra firme*	14.5 - 29.7	2
*Manilkara bidentata*	Sapotaceae	White sand	14.5 - 20.2	3
*Pouteria caimito*	Sapotaceae	River terrace	11.1 - 16.2	3
*Pradosia schomburgkiana*	Sapotaceae	White sand	14.6 - 16.2	2
*Simarouba amara*	Simaroubaceae	River terrace	37.0 - 44.2	3
*Rinorea guianensis*	Violaceae	*Terra firme*	16.0 - 17.9	2

We measured VI *E*_c_ and leaf functional traits between November 20 - December 20, 2018 (early wet season); except for trees from *Mouriri duckeana*, *Pourouma minor*, *Protium hebetatum*, *Eschweilera coriacea*, and *E. grandiflora*, which were sampled between April 26 - May 5, 2019 (late wet season). Although isoprene emissions from Central Amazon Forest woody species vary seasonally as a function of leaf age (Alves et al., 2014, 2016; Gomes Alves et al., 2023), we assumed measurements were comparable since there was little variability in environmental variables between the two sampling periods (values of air temperature, relative humidity, photosynthetic active radiation, and precipitation relative to these periods are presented in the **Supplementary Material**; **Supplementary Table S1**), and forest canopies generally show similar fractions of mature leaves throughout the wet season (Lopes et al., 2016; Alves et al., 2018). Stem functional trait data was collected between October - December/2018, April - May/2019, and July/2019, since these traits show lower plasticity and seasonal variability.

### 2.3 Branch collection

A professional tree climber collected branches of at least 2 cm in diameter from the side of the tree crown that received direct light and hence did not contain shade-adapted leaves. Although we do not have precise information on leaf age, we avoided senescent and young leaves, as well as visibly unhealthy or damaged leaves and leaves with epiphylls. After being cut, branches were slowly lowered by rope and grabbed before touching the ground to prevent stomatal closure due to impact. Collected branches were identified, cut once again under water to prevent embolism formation in open vessels, and placed inside water bottles for transport to the field camp (where they were again cut under water to restore xylem flow before gas exchange and VI *E*_c_ measurements). Due to the logistical issues involved in obtaining leaf-level physiological and VI measurements on intact branches from a large number of tall tropical trees (> 20 m height), performing measurements on cut branches in water is a broadly adopted, necessary practice to generate results capable of resolving ecological processes (Llusia et al., 2014; Albert et al., 2018; Jardine et al., 2020; Taylor et al., 2021; Gomes Alves et al., 2022, 2023; Robin et al., 2024). Moreover, there is consistent evidence that cutting branches does not significantly compromise gas exchange or VI *E*_c_ measurements (Monson et al., 1994, 2016; Keller and Lerdau, 1999; Ghirardo et al., 2016). Finally, we selected one visibly mature and healthy leaf of the branch to measure VI *E*_c_ on-site, then we removed the branch from the water, wrapped the terminal piece in moist absorbent paper, and placed it in a closed plastic bag until VI *E*_c_ measurements were finished (~ 15:00). Branches were processed in a field lab in the same day for functional trait measurements described below.

### 2.4 Leaf and stem functional trait measurements and calculations

We measured a total of 12 leaf traits: assimilation rate per dry mass (*A*_mass_), stomatal conductance (*g*_s_), foliar nitrogen per mass (*N*_mass_), foliar phosphorus per mass (*P*_mass_), leaf dry matter content (LDMC), leaf mass per area (LMA), leaf thickness (LT), force to tear (FtT), force to punch (FtP), vein density (*Vein*_Dens_), stomatal density (*St*_Dens_), stomatal guard cell length (*St*_Lgth_); and four stem traits: wood density of stem (*WD*_st_), wood density of twig (*WD*_tw_), sapwood area per basal area (SABA) and total height per basal area (THBA). Leaf traits measured in this study are indicators of light capture and photosynthetic capacity (*A*_mass_, *g*_s_, *N*_mass_, *P*_mass_, LMA, *St*_Dens_, and *St*_Lgth_) and leaf defense and persistence (LDMC, LT, FtT, FtP, and *Vein*_Dens_); while stem traits measured are related to biomechanical support, defense (*WD*_st_, *WD*_tw_, and THBA), and tolerance to drought (SABA) (Costa et al., 2018).

All functional trait measurements and calculations were performed according to published protocols (Pérez-Harguindeguy et al., 2013). We separated 3 leaves (including the leaf used to measure VI *E*_c_) to measure leaf fresh and dry mass, leaf thickness (LT), and leaf area (LA). For the remaining leaf traits, we gathered a total of 8 leaves: 3 to measure force to punch (FtP), 3 to measure force to tear (FtT), and 2 for anatomical measurements (vein density, *Vein*_Dens_; stomatal density, *St*_Dens_; and stomatal guard cell length, *St*_Lgth_). We weighed the leaves to obtain fresh mass and used a micrometer to measure LT and a table scanner to measure LA. Leaves were dried in an oven for 72 h at 60 °C and then weighed again to obtain dry mass. We measured FtP using a Pesola^®^ scale (Medio spring scale, item n. 40300) modified with a pressure set (accessory for Medio-scales, item n. 4.004), and FtT using a tearing apparatus (Hendry and Grime, 1993). FtP and FtT were quantified in N mm^-1^, considering that 1 kg of force is equivalent to 9.81 N; then FtP (N mm^-1^) = ((FtP (g)/1000) * 9.81)/5.35 (mm) and FtT (N mm^-1^) = FtT (kg) * 9.81/20 (mm) (Pérez-Harguindeguy et al., 2013). We analyzed images of scanned leaves with ImageJ software (Schneider et al., 2012) to obtain LA. We calculated specific leaf area (SLA) as the ratio of LA to leaf dry mass. We did not include petioles in the SLA calculation since they can be quite large for rainforest woody species and are usually more related to leaf positioning rather than biomass efficiency (Poorter et al., 2018). We calculated leaf dry matter content (LDMC) as the ratio of leaf dry mass to fresh mass, and leaf mass per area (LMA) as 1/SLA. Leaves used for LMA measurements were later ground using a portable blade mill, and their nutrient content was analyzed. Leaves set aside for anatomical measurements were cut in the median region, and the pieces were placed in plastic containers filled with FAA solution (Formaldehyde 37%, Glacial Acetic Acid, and Ethanol 70%) (Johansen, 1940) for 24 h, which was afterward replaced by Ethanol 70%. We processed stomatal density (*St*_Dens_), stomatal guard cell length (*St*_Lgth_), and vein density (*Vein*_Dens_) at the Biodiversity and Functional Ecology Lab (National Institute for Amazonian Research - INPA) following established protocols (Johansen, 1940). Leaf nutrient content analysis was conducted by the Soil and Plant Thematic Lab (LTSP, INPA), following established protocols (EMBRAPA, 1999), and resulted in values of phosphorus (*P*_mass_) and nitrogen (*N*_mass_) per leaf dry mass. For compound leaves, we considered a leaflet as the laminar unit for all leaf measurements described above.

With an increment borer (diameter of 5.15 mm), we punctured the tree stem at 1.3 m height and penetrated the wood until the borer reached a depth of about half of the stem diameter (at 1.3 m height). Afterward, we extracted a core wood sample to measure fresh and dry mass, fresh volume, and active xylem depth. We cut and removed the bark of a 5 cm terminal piece of twig to measure the fresh and dry mass and fresh volume. We weighed twig and stem wood samples to obtain fresh mass, then measured fresh volume using the water displacement method. Wood samples were dried in an oven for 72 hrs at 105°C and weighed to obtain dry mass. We estimated the wood densities of twig (*WD*_tw_) and stem (*WD*_st_) as the ratio of wood dry mass to fresh volume. We estimated sapwood depth using a direct method of light transmission through the wood core sample (Cosme et al., 2017; Quiñonez-Piñón and Valeo, 2018). We placed the dried stem wood sample above a direct light source and, with a magnifying glass, observed and measured with a caliper the extension of open vessels throughout the length of the sample from cambium to pith. This method assumes that there is a higher concentration of tyloses in the heartwood than in the sapwood of angiosperm trees (Déjardin et al., 2010), making it possible to differentiate these parts of the xylem based on visual methods (Pfautsch et al., 2012). We obtained tree sapwood area (SA) by subtracting the heartwood area (area of extension of closed vessels) from the total basal area (BA), and then the ratio of sapwood area to basal area (SABA) was calculated. We measured the total height of the tree (TH) with the help of a tree climber, who placed a measuring tape on the highest point of the tree canopy and extended it until it reached the ground. With this value, we calculated the proportion of total height to the basal area (THBA).

### 2.5 Leaf volatile isoprenoid emission capacity analysis

We collected air samples for VI *E*_c_ measurements on-site using a LI-6400XT gas exchange portable system (LiCor, USA). We installed a hydrocarbon filter (Restek Pure Chromatography, Restek Corporations, USA) at the air inlet of the LI-6400XT to remove VIs from incoming ambient air. All tubing in contact with the sampling air was PTFE to avoid the release of VIs. Before each measurement, we obtained a chamber blank sample from the empty leaf chamber. We separately enclosed the leaf (for compound leaves we considered a leaflet as the laminar unit) in the leaf chamber under standard conditions (Photosynthetic photon flux density (PPFD) = 1000 μmol m^-2^ s^-1^, leaf temperature = 30 °C) until net assimilation (*A*_n_), stomatal conductance (*g*_s_) and internal CO_2_ concentration (*C*_i_) were stable. The stability criterion for measurements was assigned up to one standard deviation of the mean *A*_n_, and we visually monitored *A*_n_ until values reached a plateau, beginning measurements when the instrument had reached the plateau and the defined stability criterion. The flow rate of air going into the leaf chamber was 400 μmol s^-1^, and CO_2_ and H_2_O concentrations were 400 μmol mol^-1^ and 21 mmol mol^-1^ (relative humidity of ~60%), respectively. An air sampling pump (GilAir^®^ Plus, Levitt Safety, Canada), positioned downstream, routed air exiting the LI-6400XT leaf chamber to fill adsorbent cartridges (stainless steel tubes filled with Tenax TA and Carbograph 5 TD adsorbents) at a rate of 200 ml min^-1^ for 10 min, resulting in 2 L air samples for compound identification and quantification analyses in the lab. VIs accumulated in the adsorbent cartridges were determined by laboratory analysis at the Chemical Analysis Lab of the University of Amazonas State (UEA) immediately after each VI measurement campaign.

The cartridges were analyzed with a thermal desorption system (TD; Markes International, UK) interfaced with a gas chromatograph-mass spectrometer and flame ionization detectors (GC-MS-FID; 7890B-GC and 5977A-MSD series, Agilent Technologies, USA). We loaded the cartridges in the TD automatic sampler (TD-100, Markes International, UK), which connects to the thermal desorption system. Then, the collected samples were dried by purging for 5 min with 50 ml min^-1^ of ultrahigh-purity helium (all flow vented out of the split vent) before being transferred (300°C for 10 min with 50 sccm of ultrapure nitrogen) to the thermal desorption cold trap held at -10°C (Unity Series 1, Markes International, UK). During GC injection, the trap was heated to 300°C for 3 min while backflushing with a carrier gas (helium) at a flow rate of 6 ml min^-1^ directed into the column (HP-5, 5% phenyl methyl siloxane capillary, 30.0 m x 320 μm x 0.25 μm, Agilent Technologies, USA). The oven ramp temperature was programmed with an initial hold of 6 min at 27°C, followed by an increase to 85°C at 6°C min^-1^, followed by a hold at 200°C for 6 min.

The GC-MS-FID was calibrated by injecting different amounts of gas standards into separate cartridges. The gas standard composition is shown in the **Supplementary Material** (**Supplementary Table S2**) (Apel & Riemer Environmental Inc., USA). The monoterpene composition of the cylinder reflected the most commonly observed monoterpenes emitted by Amazonian woody species (Jardine et al., 2015, 2017) which are also the most representative in terms of emissions at an ecosystem scale (Yáñez-Serrano et al., 2018). Calibration curves were carried out at least three times before the analysis of the sample cartridges, to get a mean correlation coefficient >= 0.98 (**Supplementary Figure S2**). In addition, two standard cartridges were analyzed every 20 samples to check system sensitivity. We identified isoprene and monoterpenes found in sample cartridges by comparison of observed retention times with the retention times of standards used for calibration. We identified and quantified compounds using Agilent Enhanced ChemStation (MSD ChemStation F.01.01.2317, Agilent Technologies, USA). We were not able to perform calibration curves for α-Terpinene and p-Cymene, hence, α-Terpinene and p-Cymene found in our samples were calibrated with α-Pinene.

Concentration was determined using the sample volume that was passed through each cartridge. This volume is the integration of the mass flow rate measured and controlled by the pump used to suck the air coming out from the LI-6400XT leaf chamber. Once the volume mixing ratio of VI (ppbv) was obtained, VI emission capacity per area (*E*_c,A_) was determined using the equation (*F = R*ppbv × Q/S), where *F* (nmol m^-2^ s^-1^) is the leaf flux of VIs; *Rppbv* (nmol mol^-1^) is VI concentration of the sample; Q is the flow rate of air into the leaf chamber (400 x 10–^6^ mol s^-1^); S is the area of leaf within the chamber (0.0002 and 0.0006 m²). Values of *E*_c,A_ for each monoterpene observed in samples were added to obtain the sum of monoterpene *E*_c,A_ (from now on monoterpene *E*_c_). Isoprene and monoterpene *E*_c,A_ were transformed to emission capacity per dry mass (isoprene and monoterpene *E*_c,M_) and *A*_n_ to photosynthesis per leaf dry mass (*A*_mass_), all expressed in units of *μ*g C g^-1^ h^-1^ to reflect carbon allocation dynamics in our analyses. We performed VI *E*_c_ measurements between 8:00 and 14:00 and measured trees from the same species in sequence, to reduce the effects of the plant’s physiological circadian rhythm in the results. We did not see a significant variation between the time of measurements and *A*_n_ or *g*_s_ (**Supplementary Figure S3**), which suggests that a potential midday depression in stomatal conductance did not have a strong effect on our measurements.

### 2.6 Statistical analysis

We performed principal component analyses (PCA) with scaling to ordinate trees and species based on their functional trait values and to extract, from the multivariate space of trait correlations, the first two axes that captured most trait variation (PC1 and PC2). After that, we performed mixed effects linear regression models with species as a random factor to evaluate how PC1 and PC2 scores varied between vegetation types: PC1/PC2 ~ vegetation type + (1|Species). To evaluate if there were differences between PC1 and PC2 scores of trees from species with the capacity to emit isoprene or monoterpenes (0: non-emitter; 1: emitter), we performed mixed effects linear regression models with species as a random factor: PC1/PC2 ~ capacity to emit isoprene/monoterpenes + (1|Species).

Given that the capacity to emit isoprene or monoterpenes could be limited by both ends of the fast-slow spectrum (H1), we tested whether emitters were significantly positioned (lower variance) on the PC axes than non-emitters by producing bootstrapped distributions of variance differences (non-emitter variance minus emitter variance) based on iterative random sampling of PC values from each group (emitter/non-emitter) with replacement. To test whether PC1 and PC2 scores of trees from species with the capacity to emit isoprene or monoterpenes (0: non-emitter; 1: emitter) varied between vegetation types, we performed mixed effects linear regression models with species as a random factor: PC1/PC2 scores of isoprene/monoterpene emitters ~ vegetation type + (1|Species); and PC1/PC2 scores of isoprene/monoterpene non-emitters ~ vegetation type + (1|Species).

To compare magnitudes of isoprene and monoterpene *E*_c_ between vegetation types, we performed mixed effects linear regression models with species as a random factor: isoprene *E*_c,M_/monoterpene *E*_c,M_ ~ vegetation type + (1|Species). To evaluate which variables (PC1, PC2, or vegetation type) best explained variations in the magnitudes of isoprene and monoterpene *E*_c_, we performed a stepwise model selection analysis. Statistical analyses of the magnitudes of isoprene and monoterpene *E*_c_ were always performed with log-transformed + 1 values of isoprene and monoterpene *E*_c,M_, and only included individual trees from species with the capacity to emit isoprene and/or monoterpenes.

To test if there was a tradeoff in the capacity to emit isoprene or monoterpenes, we performed a chi-squared (χ²) analysis to compare observed and expected occurrences of species that only emitted isoprene, only emitted monoterpenes, emitted both isoprene and monoterpenes, or did not emit either isoprene or monoterpenes. Lastly, we performed a mixed effects linear regression model with species as a random factor to test if there was a tradeoff in the magnitudes of isoprene and monoterpene *E*_c_ in trees from species with the capacity to emit both compounds: isoprene *E*_c,M_ ~ monoterpene *E*_c,M_ + (1|Species).

Because isoprene and monoterpene synthase encoding genes should be conserved at the taxonomic species level (Dani et al., 2014; Loreto and Fineschi, 2015), trees that did not show detectable *E*_c_ but belonged to species with the capacity to emit isoprene/monoterpenes were assigned as 1/”Yes” in mixed effects models comparing emitters and non-emitters. Similarly, we included 0 values from trees that did not show detectable *E*_c_ but belonged to species with the capacity to emit isoprene/monoterpenes in mixed effects models comparing magnitudes of isoprene and monoterpene *E*_c_. We chose this statistical approach because an absence of detectable *E*_c_ in plants from emitting species can be attributed to high intraspecific variability in enzyme activities and precursor concentrations at the MEP pathway, which has been shown to lead to differences up to seven times between different plants of the same species even when grown under the same conditions (Lehning et al., 1999; Owen and Peñuelas, 2013; Zeng et al., 2024).

Mixed effects linear regression models were performed using the lmer function of the LME4 R package (Bates et al., 2015), and results are presented as plots from the GGPLOT2 package (Wickham, 2016). The p-values of pairwise comparisons between vegetation types and linear regressions were obtained with the EMMEANS package (Lenth, 2024). All statistical analyses were performed using R version 4.3.2 through the platform RStudio 2023.9.1.494 (R core team, 2023).

## 3 Results

**Figure 1:**
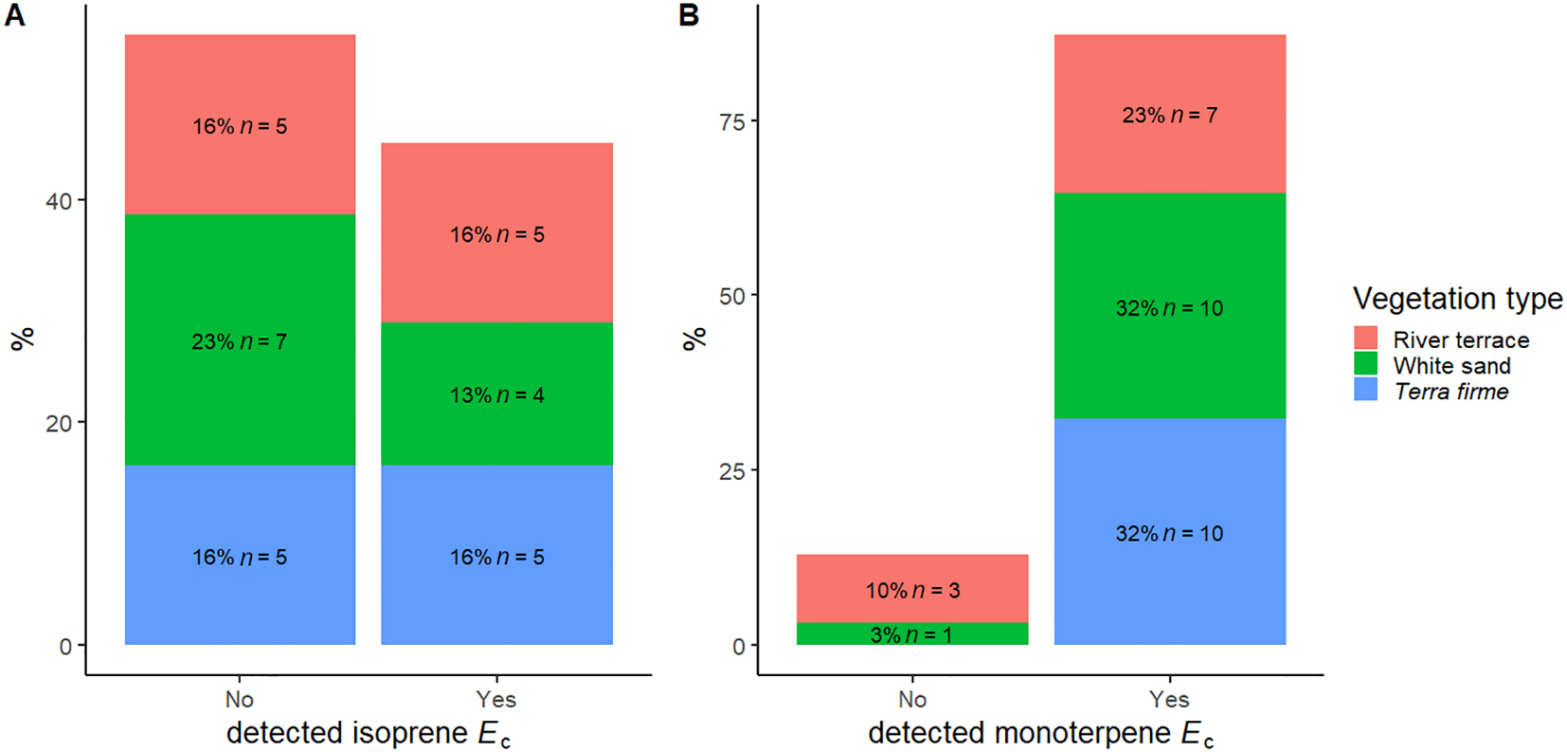
Distribution of the capacity to emit isoprene **(A)** and monoterpenes **(B)** in 31 species of angiosperm distributed across different vegetation types in the Central Amazon Forest: *Terra firme*, non-flooded upland forest; White sand, white sand forest, and River terrace, ancient non-flooded river terrace forest.

We observed the capacity to emit isoprene in 39 trees from 14 species (45% of species) and to emit monoterpenes in 49 trees from 27 species (87% of species) (**Table 2**, **Figure 1**). The most frequent emitted monoterpenes were α-terpinene (34 trees), followed by β-pinene (16 trees) and β-phellandrene (12 trees), but we also saw trees emitting camphene (3 trees), α-pinene (2 trees), p-cymene (2 trees), and β-myrcene (1 tree) (**Supplementary Table S3**). The average intra-specific variation in the magnitudes of VI *E*_c_ was lower than the inter-specific variation (**Table 2**). Magnitudes of isoprene *E*_c_ varied from 2.1 - 54.3 µgC g^-1^ h^-1^, with one tree from *Swartzia reticulata* emitting 150 µgC g^-1^ h^-1^, and of monoterpene *E*_c_ varied from 0.2 - 32.1 µgC g^-1^ h^-1^. Complete names, units, standard deviation, range in dataset, and average percentages of intra- and inter-specific variation in VI *E*_c_ and functional traits are presented in **Table 2**. Average values per species and percentages of intra-specific variation are available in the **Supplementary Material (Appendix 1)**. Species that emitted and did not emit isoprene were similarly distributed between different vegetation types, but *terra firme* did not contain non-emitters of monoterpenes (**Figure 1**). Chi-squared (χ²) analysis of observed and expected occurrences of species that only emitted isoprene, only emitted monoterpenes, emitted both isoprene and monoterpenes, or did not emit either isoprene or monoterpenes was not statistically significant (**Table 3**).

**Table 2:** Complete names, units, values of mean, standard deviation (SD), range in dataset, average (avg) intra-specific variation, and inter-specific variation of magnitudes of isoprene and monoterpene *E*_c,A_ and *E*_c,M_, and functional traits measured for 91 trees from 31 species of angiosperm in a Central Amazon Forest.

Variable	Unit	Mean	SD	Range in dataset	avg intra-specific variation %	inter-specific variation %
Isoprene emission capacity per area (Isoprene *E*_c,A_)	nmol m^-2^ s^-1^	4.7	9.9	0 - 81.6	68.6	178.0
Isoprene emission capacity per dry mass (Isoprene *E*_c,M_)	µgC g^-1^ h^-1^	8.8	18.8	0 - 150.9	70.4	182.3
Sum of monoterpene emission capacity per area (Monoterpene *E*_c,A_)	nmol m^-2^ s^-1^	0.6	1.3	0 - 7.3	104.1	118.0
Sum of monoterpene emission capacity per dry mass (Monoterpene *E*_c,M_)	µgC g^-1^ h^-1^	2.6	5.4	0 - 32.1	104.9	124.0
Net assimilation rate (*A*_n_)	µmol m^-2^ s^-1^	5.8	3.4	0.13 - 20.6	31.8	55.4
Assimilation rate per dry mass (*A*_mass_)	µgC g^-1^ h^-1^	2 130.6	1 377.7	51.2 - 8998.5	30.2	63.3
Stomatal conductance (*g*_s_)	mol m^-2^ s ^-1^	0.1	0.07	0.01 - 0.3	38.3	51.0
Foliar nitrogen per mass (*N*_mass_)	mg g^-1^	16.2	5.3	7.55 - 32.9	8.4	32.9
Foliar phosphorus per mass (*P*_mass_)	mg g^-1^	0.7	0.2	0.39 - 1.4	11.9	26.6
Leaf dry matter content (LDMC)	mg g^-1^	464.0	66.5	318.3 - 650.4	7.8	12.6
Specific leaf area (SLA)	cm^2^ g^-1^	88.0	29.3	42.6 - 197.2	12.2	30.7
Leaf mass per area (LMA)	g cm^-2^	0.01	0.004	0.005 - 0.02	12.2	30.7
Leaf thickness (LT)	mm	0.3	0.09	0.2 - 0.6	9.4	32.6
Force to Tear (FtT)	N mm^-1^	1.0	0.6	0.2 - 2.9	18.8	62.0
Force to Punch (FtP)	N mm^-1^	0.2	0.1	0.05 - 0.5	18.2	38.8
Vein density (*Vein*_Dens_)	veins mm^-2^	8.6	2.3	1.8 - 15.0	13.9	24.6
Stomatal density (*St*_Dens_)	stomata mm^-2^	0.3	0.2	0.02 - 0.8	21.6	57.0
Stomatal guard cell length (*St*_Lgth_)	µm	12.9	9.0	5.7 - 45.3	20.2	66.2
Wood density of stem (*WD*_st_)	g cm^-3^	0.7	0.2	0.29 - 0.93	8.7	22.7
Wood density of twig (*WD*_tw_)	g cm^-3^	0.7	0.1	0.39 - 0.85	6.4	14.3
Sapwood area per basal area (SABA)	m^2^ m^-2^	0.7	0.3	0.08 - 1	14.9	36.7
Total height per basal area (THBA)	m m^-2^	657.7	417.4	57.8 - 1899.0	33.8	56.1

Percentage (%) of average intra-specific variation was calculated by averaging the observed coefficients of variation > 0 (CV, CV = (SD/mean) * 100) for each variable within each species, and of inter-specific variation was calculated as the CV of average values per species for each variable. Mean values per species and intra-specific variation are provided in the **Supplementary Material (Appendix 1)**.

**Table 3:** Contingency table and chi-squared (χ^2^) p-value of comparison between observed and expected occurrences of the capacity to emit isoprene and monoterpenes in 31 species of angiosperm in a Central Amazon Forest.

	No monoterpene *E*_c_	Monoterpene *E*_c_	χ^2^ p-value
No isoprene *E*_c_	2	15	1.0
Isoprene *E*_c_	2	12	

**Figure 2:**
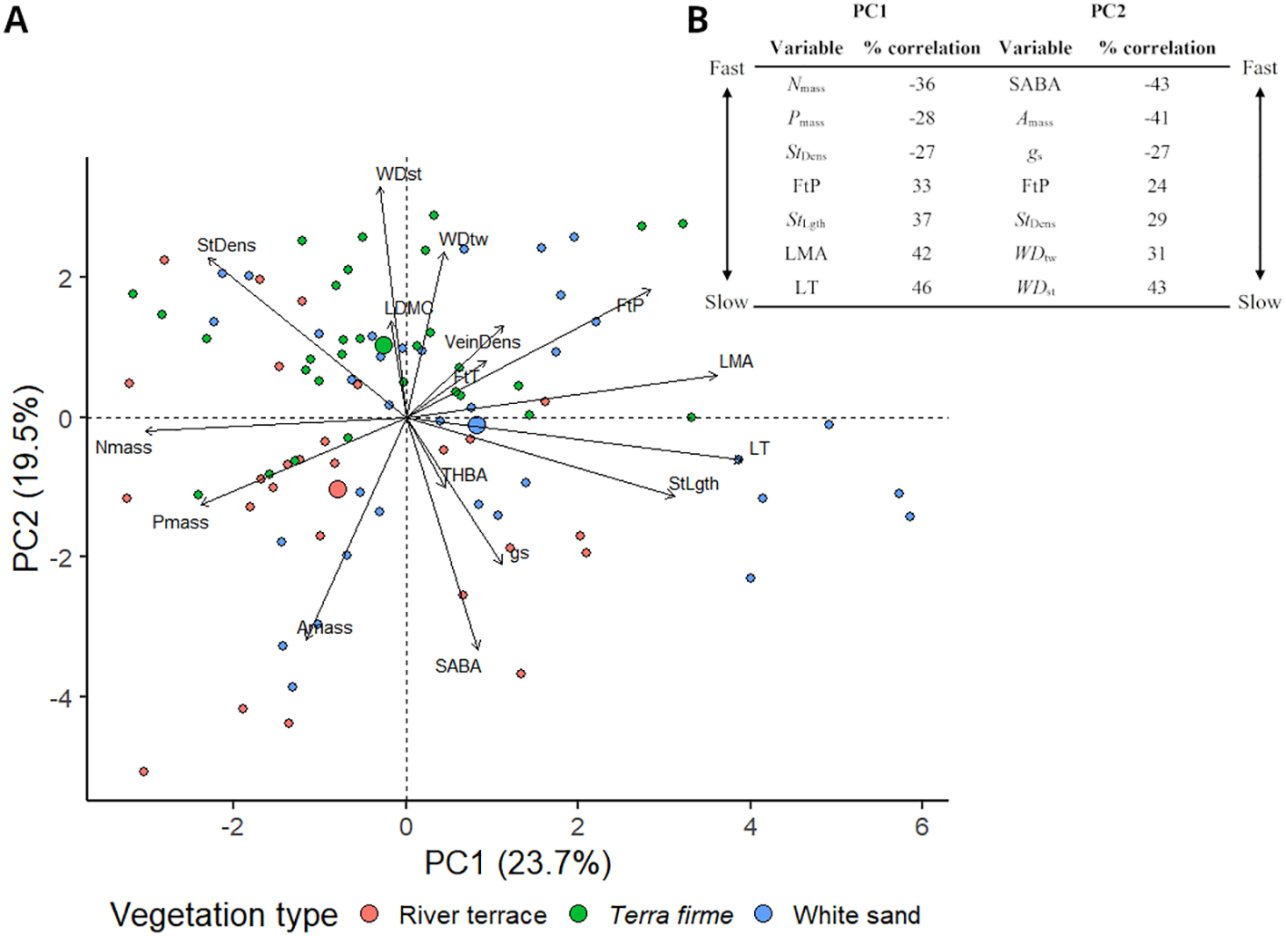
**(A)** Principal Component Analysis (PCA) of functional trait data obtained for 91 trees from 31 species of angiosperm in the Central Amazon Forest distributed across different vegetation types: *Terra firme* = non-flooded upland forest, White sand = white sand forest, and River terrace = ancient non-flooded river terrace forest. PC1 reflected a leaf economics strategy axis, while PC2 captured a mix of leaf (*A*_mass_, *g*_s_, FtP, *St*_Dens_) and stem (*WD*_st_, *WD*_tw_, SABA) strategies. Positive and negative PC scores reflected slower and faster leaf (PC1) and leaf/stem (PC2) economics strategies, respectively. Trees are labeled according to the vegetation type in which they were sampled. Big circles represent the average PCA score of the vegetation type. **(B)** Traits > 20% correlated with PC1 and/or PC2 and percentages (%) of correlation. Fast traits show a more negative % of correlation, and slow traits show a more positive one. Complete trait names are presented in **Table 2** and loadings for all principal components and scores are presented in the **Supplementary Material (Appendix 1)**.

The PCA performed with all trees revealed trait correlations that formed two main, partially independent principal component axes (PC1-2) of CES (**Figure 2A**) and, together, explained 43.2% of the variance in functional trait data. The PCA performed with species average functional trait values reflected similar correlations as the PCA performed with all trees and showed only a slightly higher percentage of variation explained by PC1 and PC2 (46.9%; **Supplementary Figure S4**). Functional traits > 20% correlated with PC1 and/or PC2 and percentages (%) of correlation are presented in **Figure 2B**. Loadings for all PC axes and scores are presented in the **Supplementary Material (Appendix 1)**.

PC1 axis showed functional trait correlations that reflected fast-slow leaf CES. Trees with more negative scores for this axis had higher *N*_mass_ and *P*_mass_, were thinner and wider (low LT and LMA), had higher densities of smaller stomata (high *St*_Dens_ and low *St*_Lght_), and weaker mechanical resistance (low FtP); thus being considered to have faster leaf strategies. Meanwhile, trees with more positive PC1 scores had lower *N*_mass_ and *P*_mass_, were thicker, narrower, and tougher (high LT, LMA, and FtP), and had lower densities of larger stomata (low *St*_Dens_ and high *St*_Lght_); thus being considered to have slower leaf strategies.

**Figure 3:**
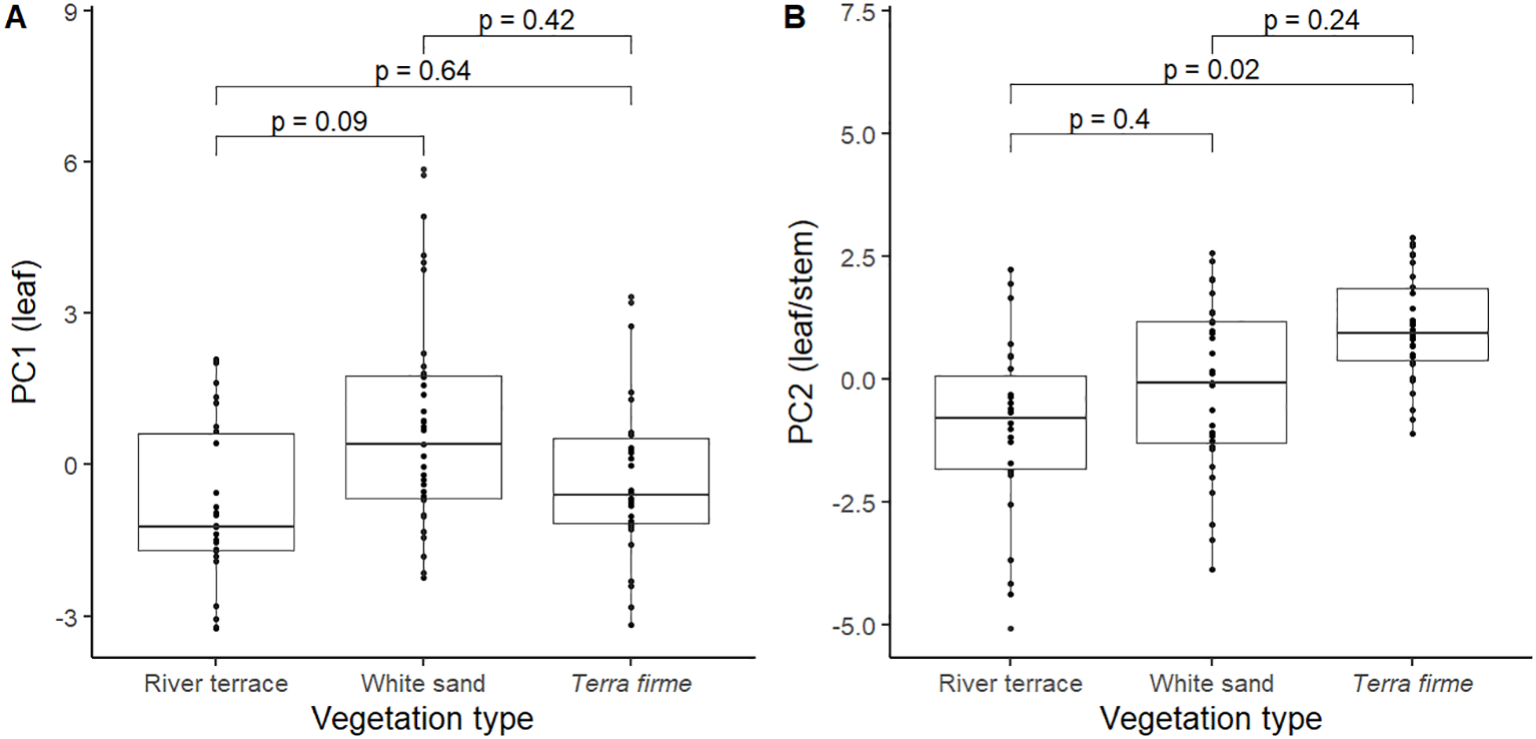
Pairwise comparisons of PC1 **(A)** and PC2 **(B)** scores between pairs of vegetation types for 91 trees from 31 species of angiosperm in the Central Amazon Forest distributed across different vegetation types: *Terra firme*; non-flooded upland forest, White sand; white sand forest, and River terrace; ancient non-flooded river terrace forest. Negative and positive PC scores reflected faster and slower leaf (PC1) and leaf/stem (PC2) economics strategies, respectively. Pairwise comparisons are mixed effects models that were performed with all trees (*n* = 91) and included species as a random factor.

PC2 axis captured a mix of leaf (*A*_mass_, *g*_s_, FtP, *St*_Dens_) and stem (*WD*_st_, *WD*_tw_, and SABA) CES. Trees with more negative scores for this axis were trees with higher *A*_mass_ and *g*_s_, lower stomatal density (*St*_Dens_), weaker leaves (low FtP), and higher proportional area of active xylem vessels (SABA) and less dense wood (low *WD*_st_ and *WD*_tw_); hence being considered to have faster leaf/stem CES. Meanwhile, trees with more positive PC2 scores had lower *A*_mass_ and *g*_s_, higher stomatal density (*St*_Dens_), tougher leaves (high FtP), and lower proportional area of active xylem vessels (SABA) and denser woods (high *WD*_st_ and *WD*_tw_); hence being considered to have slower leaf/stem CES. Pairwise comparisons did not show significant differences in PC1 values between vegetation types (**Figure 3A**) but revealed that trees from *terra firme* showed significantly slower leaf/stem CES strategies (more positive PC2 scores) compared to trees from river terrace (**Figure 3B**).

**Figure 4:**
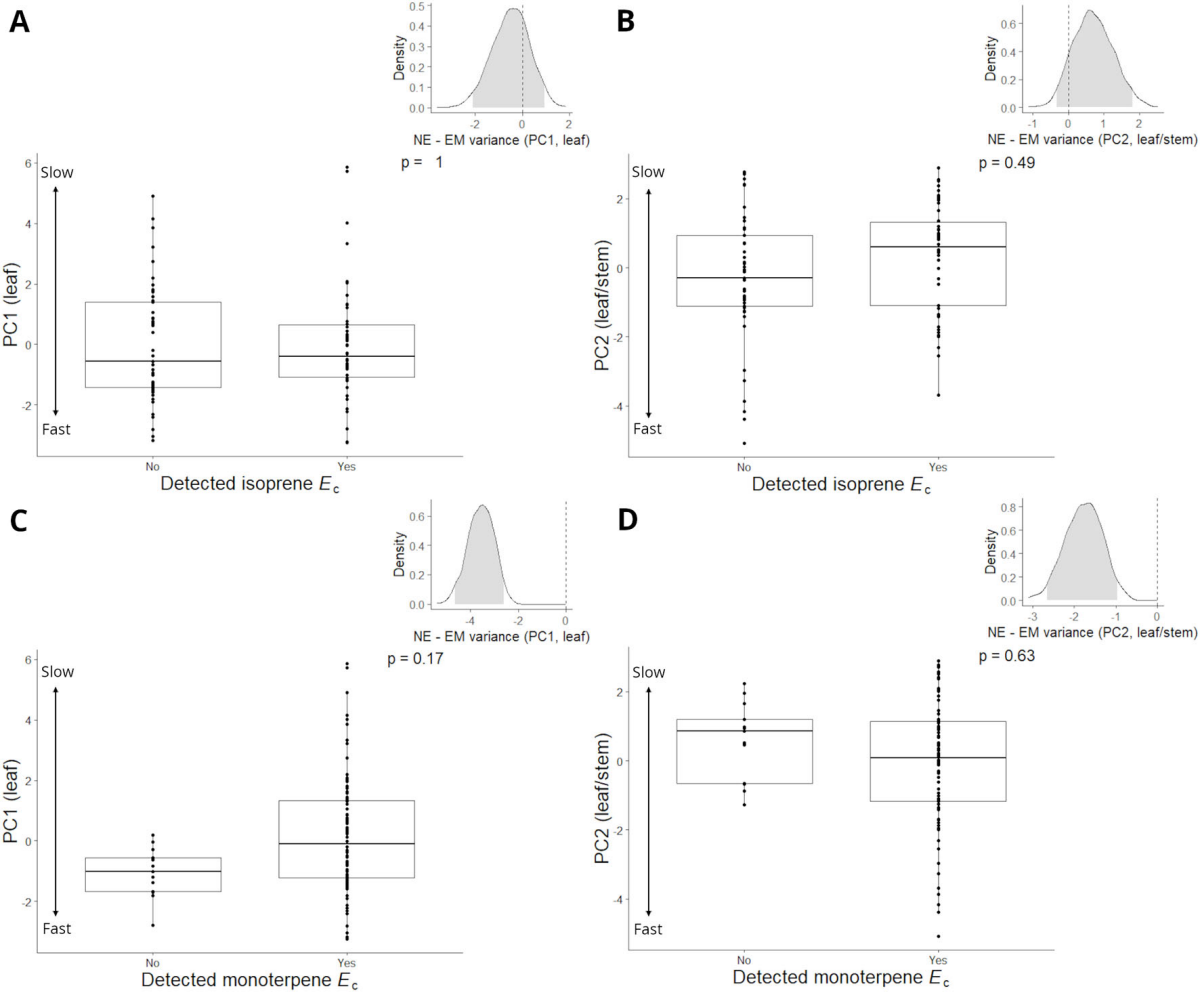
Pairwise comparisons of isoprene emitter and non-emitter (Detected isoprene *E*_c_; Yes, No) PC1 **(A)** and PC2 **(B)** scores, and monoterpene emitter and non-emitter (Detected monoterpene *E*_c_; Yes, No) PC1 **(C)** and PC2 **(D)** scores for 91 trees from 31 species of angiosperm in the Central Amazon Forest. Negative and positive PC scores reflected faster and slower leaf (PC1) and leaf/stem (PC2) economics strategies, respectively. Pairwise comparisons are mixed effects models that were performed with all trees (*n* = 91) and included species as a random factor. Inset graphs show density distributions of bootstrapped resampled variances of average PC1/PC2 non-emitter (NE variance) minus emitter (EM variance) scores for isoprene/monoterpenes. Density distribution analyses were performed with species averages (*n* = 31) of PC1 and PC2 scores.

We did not observe significant differences in mean values of PC1/PC2 between emitters and non-emitters of isoprene or monoterpenes (**Figure 4**). However, bootstrapped analyses comparing PC score variances between emitters and non-emitters showed that isoprene emitters tended to be positioned (lower PC2 variance, inset **Figure 4B**) at the slower end of leaf/stem CES (positive PC2 scores) compared to non-emitters of isoprene (**Figure 4B**). This analysis also revealed that monoterpene emitters occupied the entire range of PC1 and PC2 values and that non-emitters of monoterpenes were rare and significantly positioned (lower variance, insets **Figures 4C, D**) at the faster end of leaf CES (negative PC1 scores) and the slower end of fast/stem CES (positive PC2 scores) (**Figures 4C, D**).

**Figure 5:**
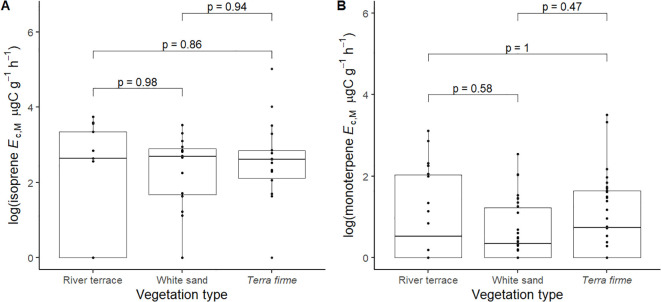
Pairwise comparisons of log + 1 of isoprene emission capacity per leaf dry mass (isoprene *E*_c,M_, µg C g^-1^ h^-1^) **(A)** and log + 1 of monoterpene emission capacity per leaf dry mass (monoterpene *E*_c,M_, µg C g^-1^ h^-1^) **(B)** between pairs of vegetation types: *Terra firme* = non-flooded upland forest, White sand = white sand forest, and River terrace = ancient non-flooded river terrace forest. Pairwise comparisons are mixed effects models that were performed with all trees from species that showed capacity to emit isoprene (**A**, *n* = 46) and monoterpenes (**B**, *n* = 78) and included species as a random factor.

Pairwise comparisons of PC1/PC2 scores between different vegetation types (**Supplementary Figure S5**) showed that non-emitters of isoprene from white sand had significantly slower leaf CES (more positive PC1) than respective non-emitters from the river terrace (**Supplementary Figure S5A**). It also showed that monoterpene emitters from *terra firme* had significantly slower leaf and stem CES (more positive PC2) than respective emitters from the river terrace (**Supplementary Figure S5D**). These are patterns that reflect the already observed distribution of CES across vegetation types (**Figure 2**), and there were no significant differences in magnitudes of isoprene and monoterpene *E*_c_ between vegetation types (**Figure 5**).

**Figure 6:**
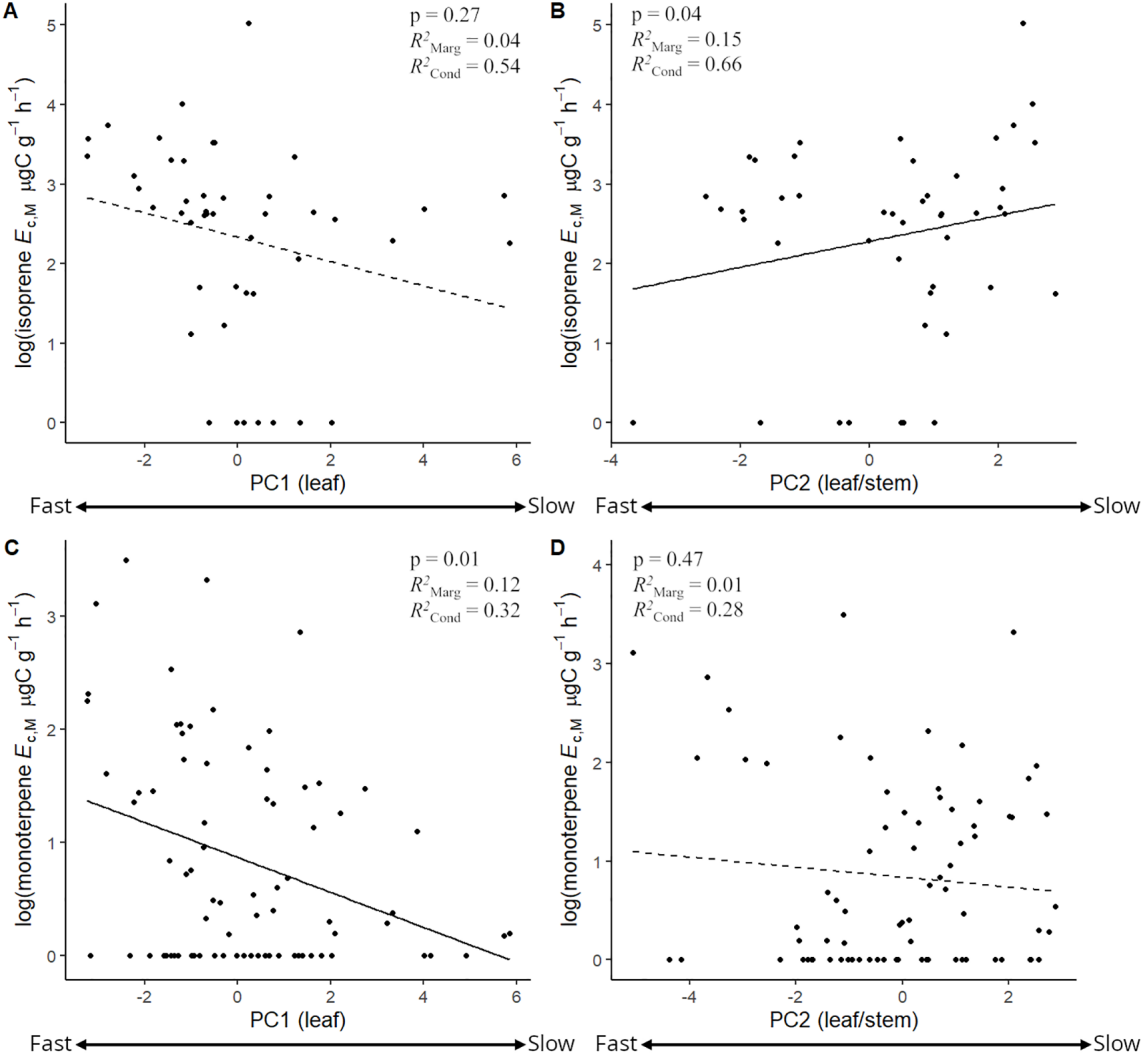
Mixed effects linear regression models between the log + 1 of isoprene emission capacity per leaf dry mass (isoprene *E*_c,M_, µg C g^-1^ h^-1^) and PC1 **(A)** and PC2 **(B)**, and between the log + 1 of monoterpene emission capacity per leaf dry mass (monoterpene *E*_c,M_, µg C g^-1^ h^-1^) and PC1 **(C)** and PC2 **(D)**. Negative and positive PC scores reflected faster and slower leaf (PC1) and leaf/stem (PC2) economics strategies, respectively. Models were performed with all trees from species that showed the capacity to emit isoprene (**A, B**, *n* = 43) and monoterpenes (**C, D**, *n* = 78) and included species as a random factor. Solid lines represent p < 0.05.

The stepwise model selection analysis (**Table 4**) revealed that PC2 and PC1 were consistently significant predictors of the magnitudes of isoprene and monoterpene *E*_c_, respectively. This analysis also confirmed that vegetation type did not have a strong effect on variation in magnitudes of isoprene and monoterpene *E*_c,_ given that the models containing only PC2 for isoprene *E*_c_ and only PC1 for monoterpene *E*_c_ had the lowest Akaike Information Criterion (AIC) (**Table 4**). Mixed effects linear regression models between magnitudes of isoprene/monoterpene *E*_c_ and PC1/PC2 (**Figure 6**) showed that isoprene *E*_c_ significantly increased toward slower leaf and stem CES (**Figure 6B**, more positive PC2 scores) while monoterpene *E*_c_ significantly increased toward faster leaf CES (**Figure 6C**, more negative PC1 scores). Finally, there was no significant relationship between the magnitudes of isoprene and monoterpene *E*_c_ in trees from species with the capacity to emit both compounds (**Supplementary Figure S6**).

**Table 4:** Stepwise model selection analysis table showing Akaike Information Criterion (AIC) and significant predictors (Sig.) of different models with increased complexity.

Independent variable (y)	Model	AIC	Sig.
log(Isoprene *E*_c,M_ + 1)	y ~ 1 + (1|Species)	142.1	
	y ~ PC1 + (1|Species)	145.1	
	**y ~ PC2 + (1|Species)**	**141.2**	* PC2
	y ~ Vegetation + (1|Species)	143.8	
	y ~ PC1 + PC2 + (1|Species)	145.3	* PC2
	y ~ PC1 + Vegetation + (1|Species)	146.9	
	y ~ PC2 + Vegetation + (1|Species)	142.6	* PC2
	y ~ PC1 + PC2 + Vegetation + (1|Species)	146.6	* PC2
log(Monoterpene *E*_c,M_ + 1)	y ~ 1 + (1|Species)	216.3	
	**y ~ PC1 + (1|Species)**	**214.6**	** PC1
	y ~ PC2 + (1|Species)	221.2	
	y ~ Vegetation + (1|Species)	219.6	
	y ~ PC1 + PC2 + (1|Species)	219.8	* PC1
	y ~ PC1 + Vegetation + (1|Species)	219.4	* PC1
	y ~ PC2 + Vegetation + (1|Species)	223.7	
	y ~ PC1 + PC2 + Vegetation + (1|Species)	223.8	* PC1

(*) p < 0.05 and (**) p < 0.01. Models with lowest AIC are in bold.

## 4 Discussion

To understand how volatile isoprenoid (VI) emissions varied with plant carbon economics strategies (CES) we present here what is, to our knowledge, the most comprehensive suite of isoprene and monoterpene emission capacity (*E*_c_) measurements, in tandem with leaf and stem functional trait data for 91 trees from 31 species of angiosperm occurring in different vegetation types (non-flooded upland *terra firme*, white sands, and ancient non-flooded river terrace forests) in the Central Amazon Forest, with many of these measurements representing the first-ever recorded data for numerous woody species. The capacity to emit monoterpenes was observed in 27 out of 31 species, and monoterpene emitters occupied the whole range of CES captured in our PCA, but magnitudes of monoterpene *E*_c_ increased toward faster leaf strategies. Meanwhile, the capacity to emit isoprene was observed in 14 species; isoprene emitters tended to be positioned toward slower leaf/stem CES, and magnitudes of isoprene *E*_c_ also increased toward slower leaf and stem CES. Finally, despite potential carbon economic limitations, the capacity to emit isoprene or monoterpenes showed no evidence of mutual exclusion, and magnitudes of isoprene and monoterpene *E*_c_ from trees of species with the capacity to emit both compounds did not vary simultaneously.

### 4.1 Principal component analysis of leaf and stem functional traits

We performed a principal component analysis (PCA) with traits measured *in situ* instead of relying on existing databases to obtain more realistic and comprehensive functional strategies for woody species prevalent in the Central Amazon Forest, many of which lacked data in current databases. Despite reflecting a smaller range of strategies than larger-scale studies (Wright et al., 2004; Kattge et al., 2011; Pérez-Harguindeguy et al., 2013; Díaz et al., 2016; Segrestin et al., 2021), our functional trait dataset contained ranges of trait values similar to those observed in other tropical studies (Baraloto et al., 2010b, 2010a; Costa et al., 2018; Vleminckx et al., 2021). We measured functional traits from species across different vegetation types to capture a broader range of CES, and trees from *terra firme* showed significantly slower leaf/stem CES compared to river terrace trees (**Figure 2**). This result could be explained by the fact that not only river terrace soils provide greater nutrient supply than *terra firme* soils, but the latter is also situated much more distant from the water table (Andreae et al., 2015) and is likely more vulnerable to drought (Costa et al., 2023) compared to the first. These are factors that possibly influenced leaf and stem traits from *terra firme* toward slower CES compared to river terrace, but more research would be needed to confirm this.

Furthermore, trees from the white sand forest had marginally significant (p = 0.09) slower leaf traits compared to trees from the river terrace, and this relationship was more evident (p = 0.04) in isoprene non-emitters. The sandy and generally nutrient-poor arenosols from white sand forests (Quesada et al., 2011) probably influenced leaf traits in this vegetation toward more resource-conservative, slow strategies (Demarchi et al., 2022). Still, this relationship was only significant for isoprene non-emitters, and this could be because, despite possible environmental drivers, river terrace trees with the capacity to emit isoprene had their trait compositions more directed toward slower leaf CES - as isoprene emitters tended to be positioned toward slower leaf/stem CES regardless of vegetation type - but the effect was not statistically significant (p = 0.26, **Supplementary Figure S7**).

Meanwhile, our analysis retrieved classic relationships from the leaf economics spectrum (Wright et al., 2004), such as positive correlations between *N*_mass_ and *P*_mass_, with both being negatively correlated with LMA. It also retrieved relationships from previously proposed wood and whole-plant economics spectrum (Chave et al., 2009; Reich, 2014), with positive correlations between *A*_mass_ and *g_s_*, both negatively correlated with wood density (*WD*_st_, *WD*_tw_). Although we measured traits in leaves from collected branches that were receiving full direct sunlight at least during part of the day and did not contain shade-adapted leaves, we understand that, given the constant fluctuations in canopy openness observed in tropical forests (Chazdon and Fetcher, 1984; Valladares et al., 2000; Montgomery and Chazdon, 2001), these leaves possibly did not develop under high light conditions as predicted by the leaf economics spectrum (Wright et al., 2004). Since this could have influenced leaf traits that are more affected by light-driven trait plasticity, we tried to overcome this issue by performing a PCA with mass-based traits (LMA, *A*_mass_, *N*_mass,_ and *P*_mass_), which are less affected by shading during leaf development (Legner et al., 2014; Keenan and Niinemets, 2016; Martin et al., 2020).

Parallel to this, *St*_Dens_ was strongly - yet opposite - correlated with both PCs. In PC1, it is possible that the negative correlation between *St*_Dens_ (*N*_mass_ and *P*_mass_) and *St*_Lgth_ (LMA and FtP) reflects a type of tradeoff where leaves with faster CES could be able to maximize CO_2_ assimilation while minimizing water loss - by having higher *N*_mass_, *P*_mass,_ and a higher density of smaller stomata (Hetherington and Woodward, 2003; Sack and Buckley, 2016). Log-scale pairwise standard major axis (SMA) regressions among these traits corroborated the directions of these relationships but showed that they sometimes differed in statistical significance between isoprene emitters and non-emitters (**Supplementary Figure S8**). For instance, only isoprene emitters showed significant relationships between *St*_Dens_, *N*_mass_, LMA, and FtP, and between *St*_Lgth_, *N*_mass_, LMA, and FtP.

At the same time, the negative correlations between *St*_Dens_ (*WD*_st_ and FtP) and *g*_s_ (*A*_mass_ and SABA) in PC2 perhaps reflected more multiple and integrated leaf/stem CES strategies. In this axis, trees with faster CES perhaps have their higher growth demands met by increased *A*_mass_, *g*_s,_ and SABA, while controlling for water loss with a decreased density of stomata. Meanwhile, trees with slower CES possibly balance carbon assimilation and mechanical/hydraulic resistance by increasing *St*_Dens_ while decreasing SABA and increasing *WD*_st_ - since narrower active xylem vessels with thicker walls (i.e., more fiber) relative to lumen area are less likely to collapse under drought (Hacke et al., 2001; Poorter et al., 2010; Hoffmann et al., 2011; Cosme et al., 2017). Log-scale pairwise SMA regressions showed that these traits followed these relationships only for isoprene emitters and were actually opposite for non-emitters, although statistical significance in these relationships for isoprene emitters was not achieved (**Supplementary Figure S9**). The integrated leaf/stem relationships observed in this second axis also corroborate the view from Reich (2014); however, trees and species were not completely aligned when comparing the first and second axes, which also corroborates the view from Baraloto et al. (2010a) that leaf and stem strategies may be decoupled in tropical forest woody species.

### 4.2 Volatile isoprenoid emissions and carbon economics strategies

Considering the idea of a balance between the benefits of VI production for plant defense and survival and the associated carbon costs of VI production, we hypothesized that (H1) the capacity to emit and magnitude of isoprene or monoterpene *E*_c_ varied with CES, and were either (H1a) associated with faster CES due to higher photosynthetic requirements to cover carbon demands of emissions, or (H1b) associated with slower strategies where higher carbon demands were compensated by greater tissue longevity. We observed the capacity to emit monoterpenes in trees distributed throughout the entire range of CES retrieved in our PCA, but the magnitudes of monoterpene *E*_c_ increased toward faster leaf strategies (supporting H1a). Meanwhile, trees with the capacity to emit isoprene were concentrated toward slower leaf/stem CES, and magnitudes of isoprene *E*_c_ also increased toward slower leaves and stems (supporting H1b). Finally, the distribution of leaf and stem strategies between vegetation types was generally consistent within emitters and non-emitters of isoprene and monoterpenes, and vegetation type was not a strong or significant predictor of variation in emission rates.

Both isoprene and light-dependent monoterpenes are produced in the methyl-erythritol 4-phosphate (MEP) pathway from recently assimilated photosynthetic carbon and serve roles in abiotic stress protection (Loreto et al., 1996; Jardine et al., 2015, 2017; Sharkey and Monson, 2017; Monson et al., 2021; Srikanth et al., 2024). Compared to isoprene, monoterpenes contain double the amount of carbon atoms in their molecules, present more complex chemical structures (e.g., acyclic, monocyclic, bicyclic) (Thulasiram et al., 2007), and are produced one chemical reaction step later (Zhao et al., 2013). However, the seemingly elevated construction costs of monoterpenes compared to isoprene might be outweighed by the fact that these compounds have additional wider ecological functions (e.g., herbivore deterrence, plant signaling), provide specialist support at different temperatures (Jardine et al., 2017; Byron et al., 2022), and are preferably produced over isoprene due to stronger enzyme affinities in the preceding steps of the MEP pathway (Harrison et al., 2013).

Even though isoprene has also been reported to influence herbivore decision (Frank et al., 2021; Pollastri et al., 2021), the extensive structural variability of monoterpene compounds may provide a significant advantage in deterring generalist herbivores and promoting the coexistence of plant species by enhancing overall chemical diversity (Salazar et al., 2018; Vleminckx et al., 2018). Therefore, monoterpene emissions should be particularly advantageous in such an ecologically complex and species-diverse tropical forest like the Amazon Forest (Asner et al., 2014; Sakschewski et al., 2016; Cardoso et al., 2017; Massad et al., 2017), which perhaps explains why the capacity to emit monoterpenes was observed in trees distributed throughout the whole range of CES (Joo et al., 2018) captured in our PCA.

Meanwhile, trees with the capacity to emit isoprene showed a tendency to be concentrated toward slower leaf and stem CES, and the magnitude of isoprene *E*_c_ also increased toward slower leaves and stems. Isoprene had been previously associated with faster leaf economics strategies due to observed relationships between higher isoprene emission rates, higher photosynthetic capacity, and shorter leaf lifespan (Harrison et al., 2013; Dani et al., 2014), in addition to its possible role as a thermoprotector and hypothesized sink of excess energy (Singsaas et al., 1997; Behnke et al., 2007; Morfopoulos et al., 2013; Pollastri et al., 2014, 2019). However, isoprene is currently viewed under a more holistic perspective, being suggested to be a metabolite that coordinates gene expression and mediates resource supply and demand in stress responses (Monson et al., 2021).

In this context, studies have recently observed relationships between the capacity to emit and the magnitude of isoprene *E*_c_ and increased accumulation of lignin and leaf toughness (Fernández-Martínez et al., 2018; Zuo et al., 2019; Monson et al., 2020, 2021; Yuan et al., 2020; Robin et al., 2024). Leaf toughness (Force to Punch, FtP) was a trait contained within the PC2 axis, suggesting that the association between isoprene emissions and increased leaf mechanical resistance may extend itself to increased stem resistance, but more research would be needed to test this hypothesis. Nevertheless, the observed association between the presence and magnitude of isoprene emission capacity and slower CES may reflect an underlying trade-off between investment in growth- and defense-related traits. More specifically, isoprene emitters perhaps prioritize structural defenses (e.g., increased leaf toughness and stem resistance) over traits linked to rapid resource acquisition (e.g., high *N*_mass_ and *P*_mass_). Such a trade-off is consistent with recent perspectives on the multifunctional role of isoprene, which highlight its involvement not only in abiotic stress tolerance but also in the modulation of defense responses (Monson et al., 2021). Therefore, it is possible that, in these Central Amazon Forest woody species, the carbon cost of isoprene production is compensated by enhanced tissue longevity and mechanical protection, supporting the hypothesis (H1b) that, for isoprene, slower-growing strategies can accommodate the metabolic costs of emissions by reinforcing plant persistence and defense capacities.

Furthermore, the fact that this second axis of mixed leaf/stem CES seemed to be more associated with the capacity to emit isoprene and also significantly predicted isoprene *E*_c_ may corroborate the view that isoprene’s role is not restricted to leaf-level stress responses and that the compound has a more systemic role in the mediation of growth and defense responses in the entire tree (Monson et al., 2021). Similarly, results from pairwise standard major axis (SMA) regressions between traits from this axis in isoprene emitters may further support this idea. Parallel to this, the observed increase in isoprene *E*_c_ with slower, more conservative leaf and stem CES may reflect an additional mechanism to cope with mild drought (Niinemets, 2010; Peñuelas and Staudt, 2010; Taylor et al., 2018), as occurs during the relatively short dry season at the ATTO site (Schmitt et al., 2023), but more research would be needed to confirm this.

On the other hand, magnitudes of monoterpene *E*_c_ significantly increased toward faster leaf CES. While the relationship between isoprene *E*_c_ and PC1 indicated a similar pattern, this was not statistically significant. In any case, isoprene and light-dependent monoterpene emissions most often incur the instantaneous use of recently assimilated photosynthetic carbon that could otherwise be allocated to respiration and growth (Delwiche and Sharkey, 1993; Affek and Yakir, 2003; Loreto et al., 2004; Huang et al., 2017; Lehmanski et al., 2024). Therefore, it is reasonable that, within isoprene and monoterpene emitters, magnitudes of *E*_c_ would increase toward faster leaves since a faster leaf metabolism - with higher photosynthetic rates - would generally be needed to provide sufficient carbon assimilation to support higher emission rates (Loreto and Sharkey, 1990; Delwiche and Sharkey, 1993; Loreto et al., 1996; Magel et al., 2006; Sharkey and Monson, 2017). However, the relationship between leaf CES and magnitudes of isoprene *E*_c_ might be more complex, as the compound is likely associated with both growth and defense responses, and a more significant relationship between isoprene and slower leaf and stem strategies is likely smoothing the effect of faster leaf strategies in supporting higher magnitudes of isoprene *E*_c_.

Finally, chi-squared (χ²) analysis comparing observed and expected occurrences of isoprene and monoterpene emitters was not significant, and *E*_c_ magnitudes of trees from species that emit both compounds did not have a significant relationship. This suggests that, in central Amazon tree species, isoprene and monoterpene emissions do not vary simultaneously (Harrison et al., 2013), and the absence of a relationship between both compounds might be a result of the “opportunistic” character of their production. The presence of isoprene and terpene synthase encoding genes is, ultimately, the determinant of isoprene and monoterpene emissions (Dani et al., 2014; Loreto and Fineschi, 2015). This is emphasized by the results of mixed effects linear regression models, which showed the importance of taxonomic species in emission variation by the overall smaller marginal R-squared (R^2Marg^) value - which represents the variance explained by fixed predictors - compared to the R-squared conditioned to the random factor (R^2Cond^) in the models. Yet, while terpene synthases are widespread, the evolution of isoprene synthase is quite puzzling, as the capacity to emit isoprene seems to appear and disappear in plant lineages without a clear phylogenetic thread (Dani et al., 2014; Loreto and Fineschi, 2015).

However, it has been suggested that isoprene synthase has evolved from terpene synthases (Sharkey et al., 2013; Li et al., 2017). Considering the high species richness and complexity of ecological interactions observed in Amazonian tree communities (Cardoso et al., 2017; ter Steege et al., 2025), and the importance of monoterpenes for plant signaling (Gershenzon and Dudareva, 2007; Fineschi and Loreto, 2012; Xiao et al., 2012), we argue that it would be highly advantageous for an isoprene-emitting species to retain terpene synthase genes and be able to produce both compounds. Moreover, the fact that we have observed many species emitting both isoprene and monoterpenes (12 species) or only monoterpenes (15 species) versus two species that only emitted isoprene and two species that did not emit any VIs further supports this, but more research would be needed to test this hypothesis. As non-emitters of monoterpenes were rare (two species), and positioned at the faster end of leaf CES and the slower end of fast/stem CES, a deeper evaluation of possible alternate defense strategies for these species (e.g., phenolics or alkaloids) would be interesting for a future study.

Lastly, although we cannot fully disentangle slow constitutive, stress-induced, and light-dependent monoterpene emissions within our measurements, we discard the possibility of having captured heat stress-induced emissions since measurements were done at a standard leaf temperature of 30°C, which is not stressful for tropical trees (Slot and Winter, 2017). Moreover, although we could have captured slow constitutive or even small indirect herbivory-induced emissions (Ton et al., 2006; Frost et al., 2007) triggered by herbivory/pathogens on other leaves from the measured tree or neighboring trees (Fineschi and Loreto, 2012), these would not be expected to increase with higher *N*_mass_ and *P*_mass_ (Kesselmeier and Staudt, 1999). Therefore, we argue that the relationship between increasing magnitudes of monoterpene *E*_c_ in faster leaves is probably being driven by light-dependent monoterpenes.

In that sense, we did not discuss sesquiterpene emissions in this study as they were not observed in our measurements. Likely, we did not observe sesquiterpene emissions because these derive solely from storage pools, being light-independent, and constitutive storage pool emissions at non-stressful standard conditions were possibly too low to be detected by our measurement system. Nevertheless, a parallel study did detect sesquiterpene emission rates from one tree species (*Protium hebetatum*) during the dry season (Gomes Alves et al., 2022), and brevideciduous isoprene emitters from *terra firme* showed higher diversity of stored sesquiterpene compounds and total phenolics content with increases in magnitudes of isoprene *E*_c_ (Robin et al., 2024). These studies further indicate the importance of sesquiterpene emissions in stress responses, as well as reinforce the idea that isoprene emissions possibly coordinate the supply of resources to the production of defense compounds (Monson et al., 2021).

### 4.3 Distribution of isoprene and monoterpene emissions

Previous synthesis studies assumed that about 20-38% of tropical woody species emitted isoprene (Harley et al., 2004; Loreto and Fineschi, 2015), but more recently, studies have revealed that this percentage can be even higher, up to 76% (Jardine et al., 2020; Mu et al., 2022). In our study, we observed the capacity to emit isoprene in 45% of the species measured, and a vast majority of species showed the capacity to emit monoterpenes (87%). Such high numbers of monoterpene emitters and emitters of both isoprene and monoterpenes reinforce the idea that monoterpene emissions were possibly preferentially selected by competitive and herbivore pressure under the great species richness and complexity of ecological interactions found in Amazonian tree communities - factors that were shown to be associated with increases in monoterpene emission rates (Kigathi et al., 2019) and in the number of monoterpene emitters (Massad et al., 2017; Sedio et al., 2017; Sun et al., 2024).

Such a great proportion of monoterpene emitters and the absence of non-monoterpene emitters in *terra firme* corroborate a consistent trend of studies revealing larger numbers of monoterpene emitters and higher monoterpene emission rates in the Amazon Forest than previously thought (Gomes Alves et al., 2022; A. B. Jardine et al., 2015; K. J. Jardine et al., 2017, 2020; Kuhn et al., 2004, 2007; Yáñez-Serrano et al., 2018). It also supports observations for a site in eastern Amazonia showing that the magnitude of monoterpene emissions could be as great as ~10% that of isoprene emissions (Sarkar et al., 2020), with even higher proportions during the El-Niño years (Pfannerstill et al., 2018). From the perspective of forest-atmosphere interactions, monoterpenes are much more chemically reactive than isoprene, with a yield rate of secondary organic aerosol (SOA) formation that can reach ~10% (Griffin et al., 1999b, 1999a) while isoprene has been reported as <6% (Kroll et al., 2005; Xu et al., 2014), indicating the importance of putting effort into better quantifying monoterpene emissions from Amazon woody species.

### 4.4 Study limitations and implications for emission modeling

The numerous logistical challenges associated with conducting extensive field campaigns in the Amazon Forest - e.g., remote and hard-to-access sites, uncertainties in taxonomic classifications, difficulties in locating sufficient repetitions per species, difficulties in sampling branches from very tall trees - contributed to limitations in the numbers of available replicates per species and of leaves measured per tree. Increasing these numbers could have helped reduce the observed intra-specific variation in VI *E*_c_ magnitude, as well as potentially increase the number of species with the capacity to emit isoprene, which could have helped better elucidate the observed relationships between isoprene and fast-slow CES. In that sense, in this study, we applied resampling techniques to robustly quantify uncertainty in small sample sizes (Efron and Tibshirani, 1994), and consistently included species as a random effect in our general linear regression models to address the non-independence among observations within the same species and partition the variance attributable to species-specific effects (Bates et al., 2015).

Nonetheless, future studies should consider employing Bayesian hierarchical models with informative priors and adjusted degrees-of-freedom methods (e.g., Kenward-Roger adjustment) to better address potential biases from limited sample sizes (Kenward and Roger, 1997). We also recommend that future research should invest in the training of specialized field taxonomists and the development of more comprehensive taxonomic inventories to reduce uncertainties in species classification and increase species sample sizes. Moreover, conducting a rapid photosynthetic pre-screening among several trees of the same species can help target VI measurements to trees within a similar photosynthetic range, which has been shown as associated with a drastic reduction of intra-specific variability in VI *E*_c_ (Zeng et al., 2024).

Still, we argue that observing isoprene or monoterpene emissions in a single leaf from a single tree is sufficient to classify the species as an emitter, as emissions should be conserved at the taxonomic species level (Dani et al., 2014; Loreto and Fineschi, 2015). The primary focus of our study was to position VI emitters along CES and evaluate variations in magnitudes of VI *E*_c_ across CES. Hence, we did not formulate hypotheses for non-emitters, and we argue that a scenario where non-emissions were incorrectly attributed to potential emitters would not affect our conclusions. Furthermore, the data and results we presented here contain valuable information to motivate further investigation on how VI emissions vary with plant functional strategies in the Amazon Forest and highlight the importance of leaf-level measurements to accurately estimate monoterpene fluxes; since measurements of monoterpene fluxes at ecosystem or canopy level usually fail to register actual emission rates due to strong photochemical oxidation of monoterpenes before they reach the above canopy (Kuhn et al., 2007; Jardine et al., 2015).

In addition, although the Amazon Forest is the largest source of VI fluxes to the global atmosphere (Guenther et al., 2012; Sindelarova et al., 2014), model estimates still carry high uncertainties because only a few observational studies with mechanistic and process-based approaches have been conducted, hindering modeling optimization (Alves et al., 2018; Yáñez-Serrano et al., 2020). Two major reasons for model uncertainties are: i) the correct determination of VI source magnitude or *E*_c_ (also known as emission factor); and ii) the determination of the tree emitter fraction of a given forest, as represented in models (e.g., MEGAN v2.1; Guenther et al., 2012) (Gomes Alves et al., 2023). For instance, MEGAN v.2 relies on very simplified schemes of plant functional type (PFT) distributions (e.g., CLM4 model), with a single *E*_c_ value for each PFT. Moreover, *E*_c_ values input in current models are generally derived either from a limited number of flux tower measurements - obtained exclusively from *terra firme* forests in the central Amazon (Alves et al., 2018; Sarkar et al., 2020; Langford et al., 2022; DiMaria et al., 2023; Gomes Alves et al., 2023) - or from flight measurements and satellite retrievals, which are often restricted to isoprene (e.g., Bauwens et al., 2016; Gu et al., 2017). In this light, this study indicates a direction to improve global VI model predictions by estimating more accurate *E*_c_ values and emitter fractions of Amazonian Forest types with the support of functional ecology.

Here, we showed that the capacity to emit and the magnitude of isoprene and monoterpene *E*_c_ differentially varied with fast-slow CES. While the capacity to emit monoterpenes was widespread throughout the whole range of CES captured in our PCA, magnitudes of monoterpene *E*_c_ significantly increased in faster leaves. Meanwhile, the capacity to emit isoprene was associated with slower leaf and stem CES, and magnitudes of isoprene *E*_c_ increased with slower leaves and stems as well. Based on our findings, we argue that, with more studies dedicated to further elucidate the relationships between plant functional strategies and VI emissions, soon we will be able to parameterize and scale up forest VI emission factors using more accurate plant functional trait distributions. This is very promising because plant functional traits are not only easier to measure than VI emissions but are also increasingly accessible through expanding open databases.

## 5 Conclusion

Our study presented a unique dataset of *in situ* isoprene and monoterpene *E*_c_, and functional trait measurements for 91 trees from 31 species of angiosperm distributed across different vegetation types in the central Amazon. We saw that VI emissions were equally distributed across vegetation types, with a surprisingly high amount of monoterpene emitters (87% of species) that showed increasing monoterpene *E*_c_ with faster leaf strategies. We also observed the capacity to emit isoprene in 45% of species, and isoprene emitters tended to be concentrated toward slower leaf and stem strategies, with isoprene *E*_c_ also significantly increasing toward slower leaves and stems. We provided a more integrated view of the relationships between VI emissions and functional traits on different organ-system levels and a direction for new studies to improve modeled emission estimates based on forest functional compositions. Our study adds a new piece to the development of a more biologically robust way of estimating plant VI emissions and reducing model uncertainties; this is particularly critical in highly biodiverse environments such as the Amazon Forest, which are considered the main source of VIs to the global atmosphere.

## Data Availability

The dataset presented in this study is available at: https://doi.org/10.17871/ATTO.364.8.1600.
