# Supplementary material for: Leaf isoprene and monoterpene emissions vary with fast-slow carbon economics strategies in central Amazon woody species
Source: Front Plant Sci. 2025 May 27;16:1561316. doi: 10.3389/fpls.2025.1561316 (PMC12149424; doi:10.3389/fpls.2025.1561316)
Supplement: Supplementary file 2 [file DataSheet2.docx]

***Supplementary Material***

**1. Supplementary Figures**

**Figure S1.** Location of the ATTO site and plots covering different vegetation types: Upland forest = dense non-flooded upland forest vegetation (locally called *terra firme*; one plot); White-sand forest = vegetation on white sands (two plots); Ancient river terrace forest = ancient non-flooded river terraces (one plot); adapted from Gomes Alves et al. (2022). Background topography is based on the Shuttle Radar Topography Mission (NASA-JPL, 2013) elevation model. The red polygon highlights the limits of the Uatumã Sustainable Development Reserve (USDR), and major rivers are labeled. Background layer of the insert map: ©OpenStreetMap contributors 2020. Distributed under a Creative Commons BY-SA License.


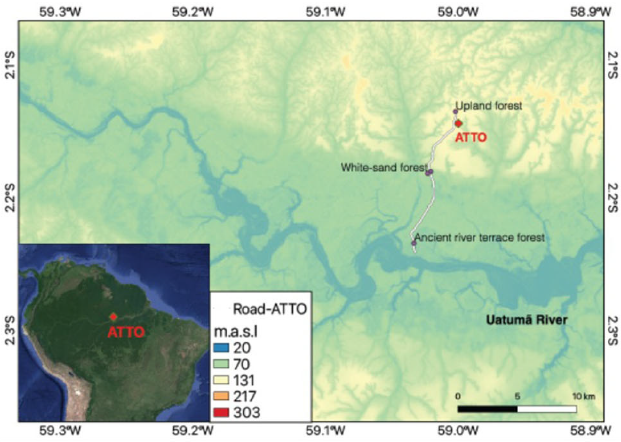


**Figure S2.** Regression lines, equations and R^2^ values of calibration curves used to quantify isoprene and monoterpenes present in our samples.

**
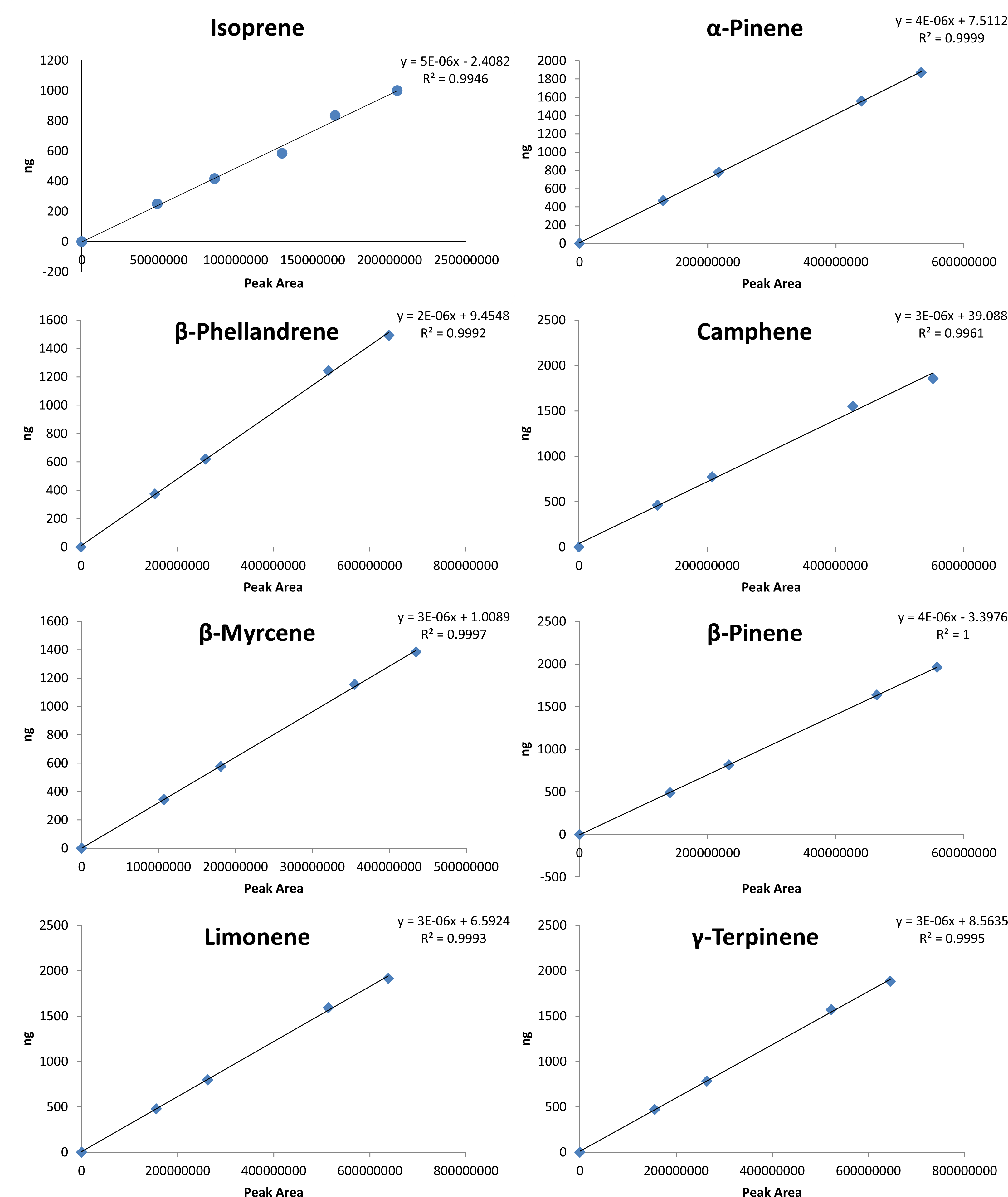
**

**Figure S3.** Values of net photosynthesis (*A*_n_, μmol m^-2^ s^-1^) (A) and stomatal conductance (*g*_s_, mol m^-2^ s ^-1^) (B) obtained at different times of the day.


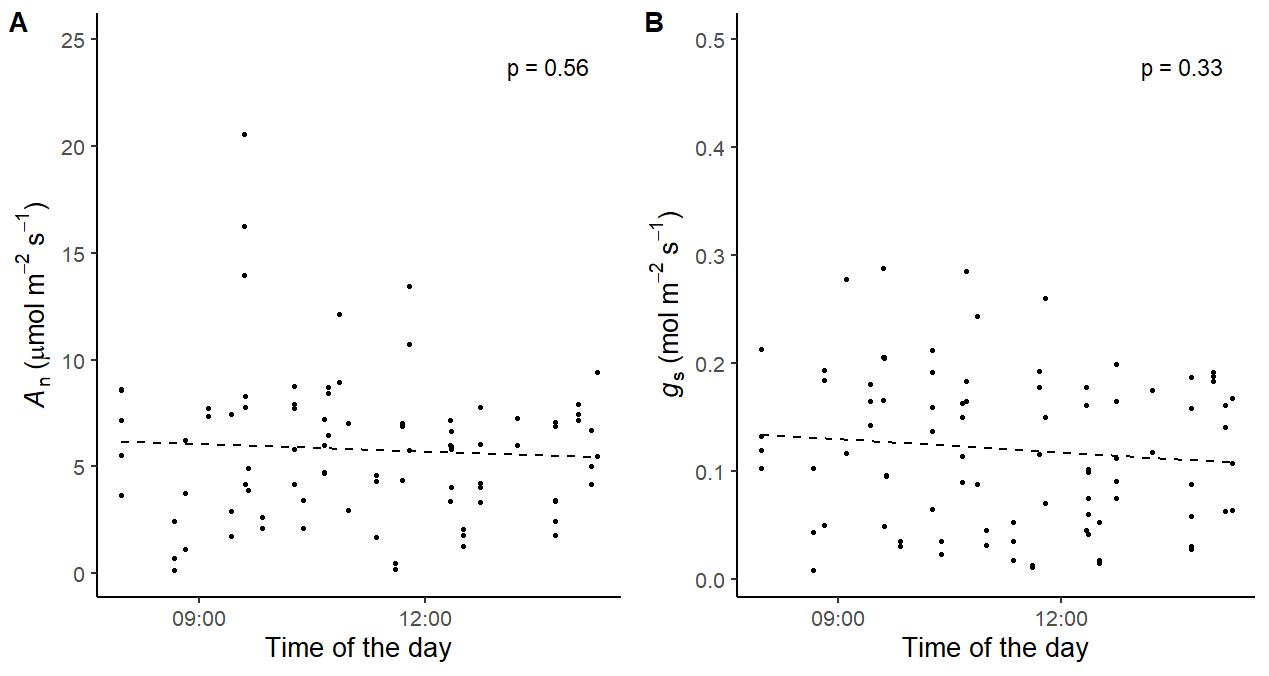


**Figure S4.** Principal Component Analysis (PCA) of functional trait data obtained for 31 species of angiosperm in a central Amazon forest distributed across different vegetation types: *Terra firme* = dense non-flooded upland forest vegetation; White sand = vegetation on white sands, and River terrace = ancient non-flooded river terraces. PC1 reflected a leaf economics strategy axis, while PC2 captured a mix of leaf (*A*_mass_, *g*_s_, FtP, *St*_Dens_) and stem (*WD*_st_, *WD*_tw_, SABA) strategies. Negative and positive PC scores reflected faster and slower leaf (PC1) and leaf/stem (PC2) economics strategies, respectively (Fig. 2B, main text). Trees are labeled according to the vegetation type in which they were sampled. Big circles represent the average PCA score of the vegetation type. Complete trait names are presented in Table 2 (main text) and loadings for all principal components and scores are presented in the supporting information (Appendix 2).


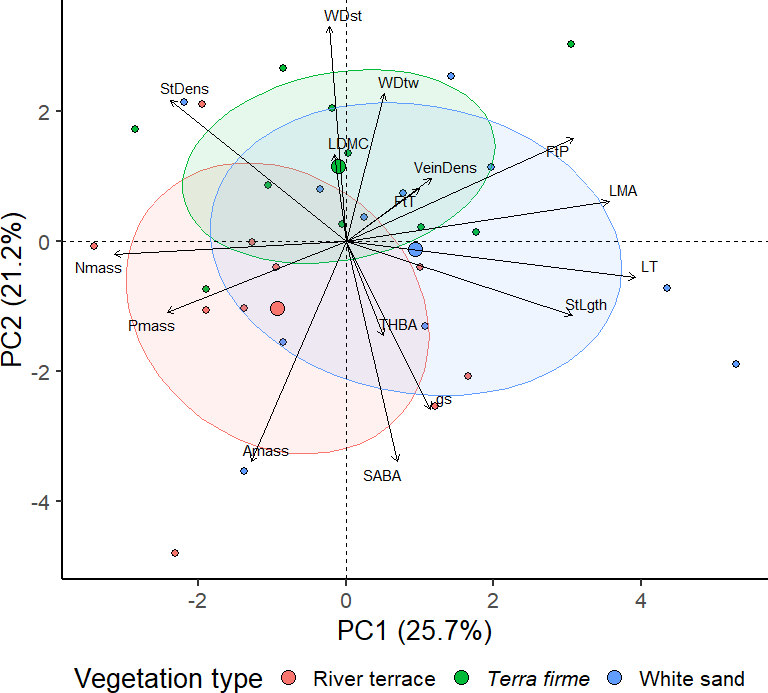


**Figure S5.** Pairwise comparisons of isoprene emitter and non-emitter (Detected isoprene *E*_c_; Yes, No) PC1 (A) and PC2 (B) scores and monoterpene emitter and non-emitter (Detected monoterpene *E*_c_; Yes, No) PC1 (C) and PC2 (D) scores between pairs of vegetation types: *Terra firme* = dense non-flooded upland forest vegetation; White sand = vegetation on white sands, and River terrace = ancient non-flooded river terraces. Negative and positive PC scores reflected faster and slower leaf (PC1) and leaf/stem (PC2) economics strategies, respectively. Pairwise comparisons are mixed effects models that were performed with all trees (*n* = 91) and included species as a random factor.

**
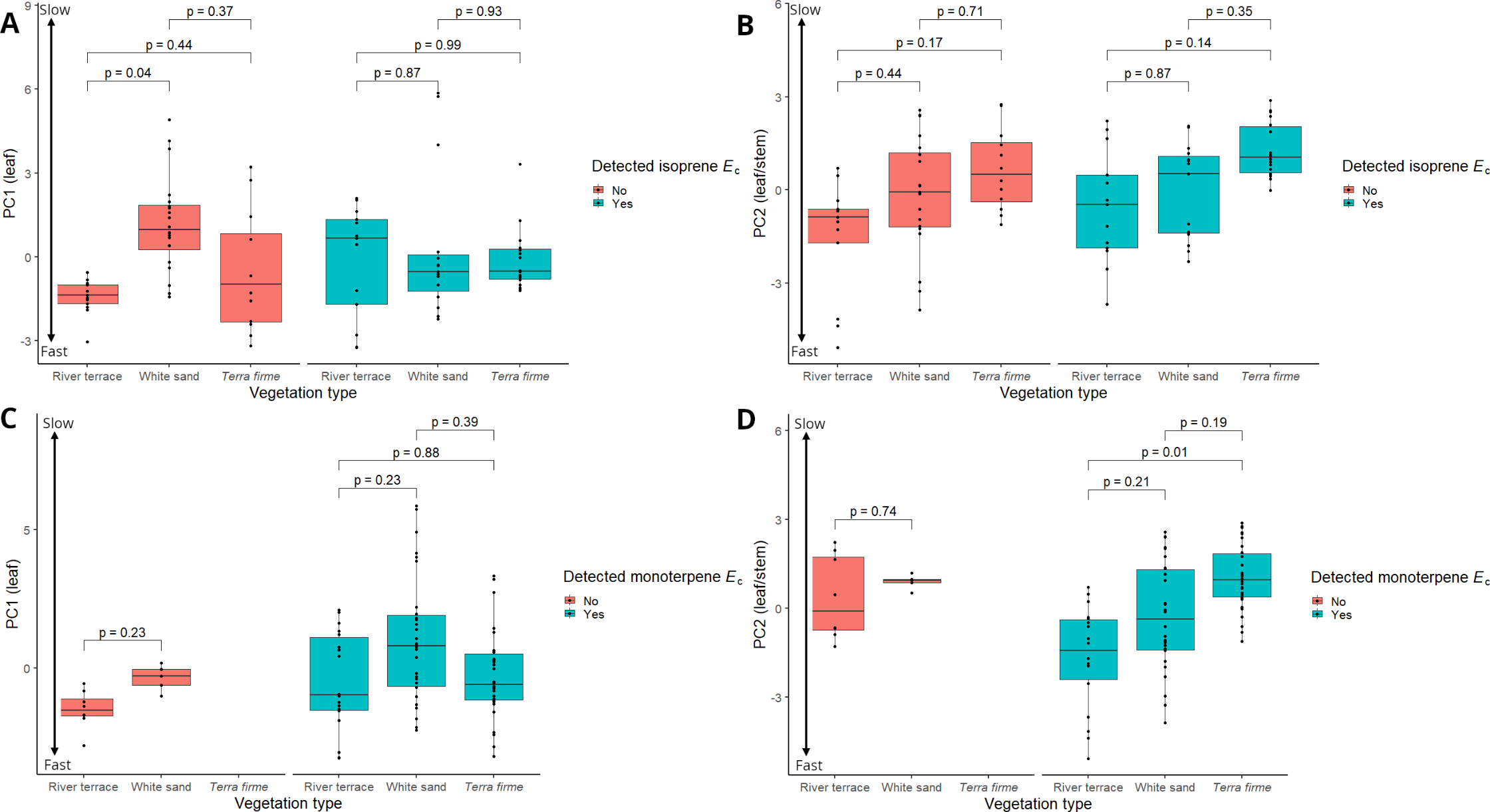
**

**Figure S6.** Mixed effects linear regression model between log +1 of isoprene emission capacity per leaf dry mass (isoprene *E*_c,M_, µg C g^-1^ h^-1^) and log +1 of monoterpene emission capacity per leaf dry mass (monoterpene *E*_c,M_, µg C g^-1^ h^-1^). The model was performed with trees from species that emitted both isoprene and monoterpenes (*n* = 38) and included species as random a factor.

**
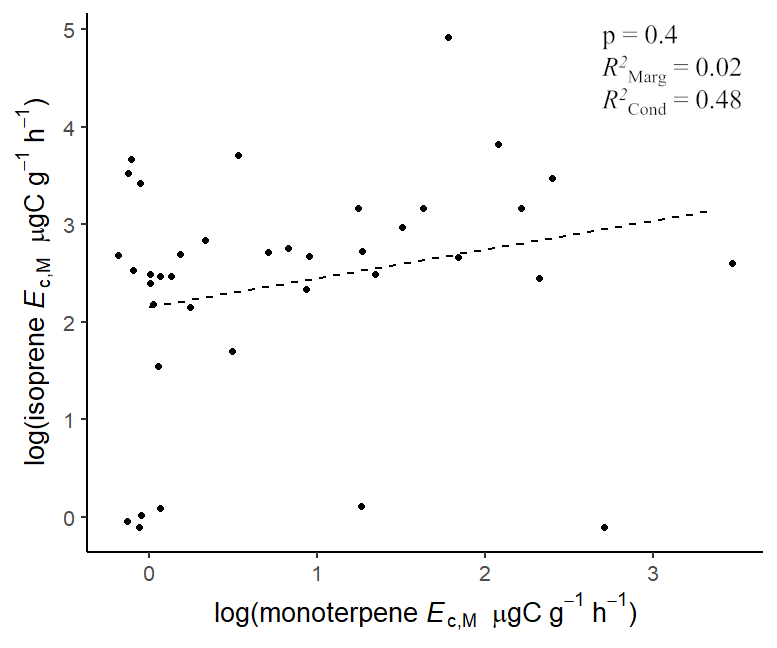
**

**Figure S7.** Pairwise comparisons of PC1 scores observed for emitters and non-emitters of isoprene (Detected isoprene *E*_c_; Yes, No), across different vegetation types: *Terra firme* = dense non-flooded upland forest vegetation; White sand = vegetation on white sands, and River terrace = ancient non-flooded river terraces. Negative and positive PC1 scores reflected faster and slower leaf economics strategies, respectively. Pairwise comparisons are mixed effects models that were performed with all trees (*n* = 91) and included species as a random factor.


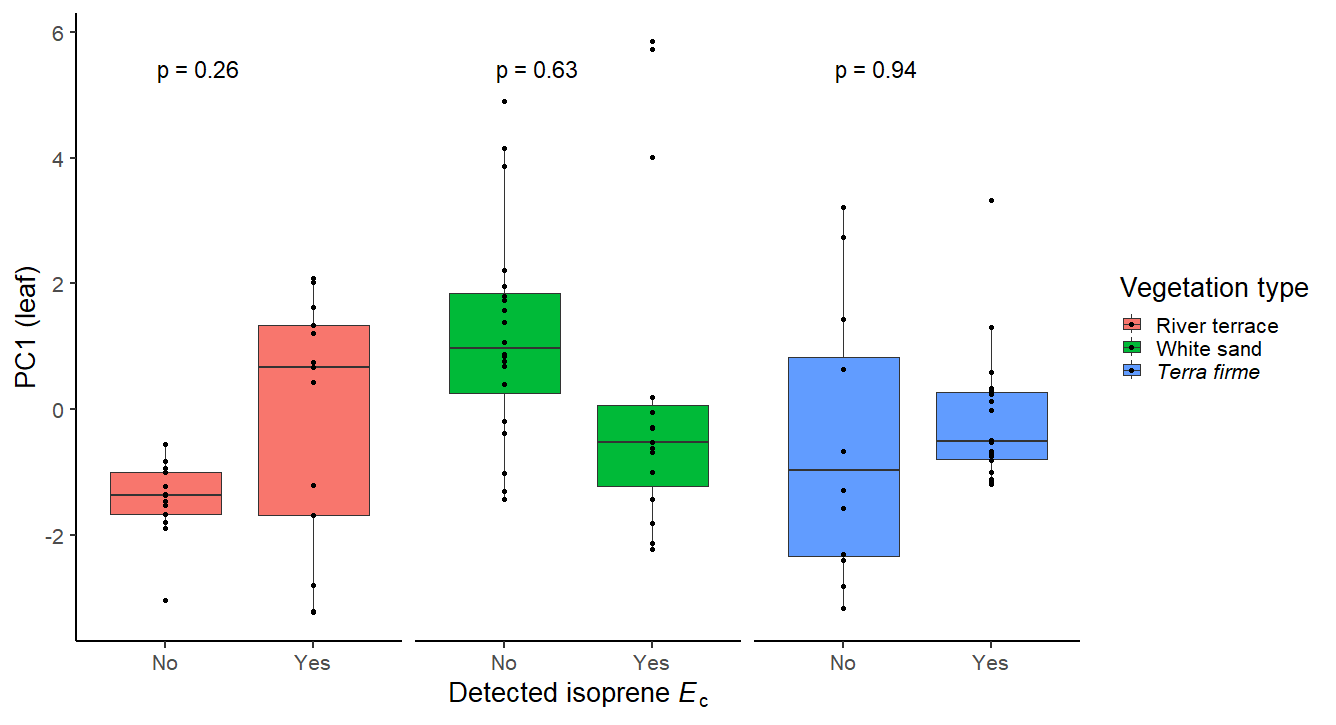


**Figure S8.** Results of log-scaled pairwise standard major axis regressions (SMA) for 91 trees from 31 species of angiosperm in a central Amazon forest performed between stomatal density (*St*_Dens_) and leaf traits which showed positive (foliar nitrogen, *N*_mass_; and phosphorus per dry mass, *P*_mass_) and negative correlations with *St*_Dens_ (stomatal guard cell length, *St*_Lgth_; leaf mass per area, LMA; force to Punch, FtP) in the first principal component extracted from the principal component analysis (PCA).

**
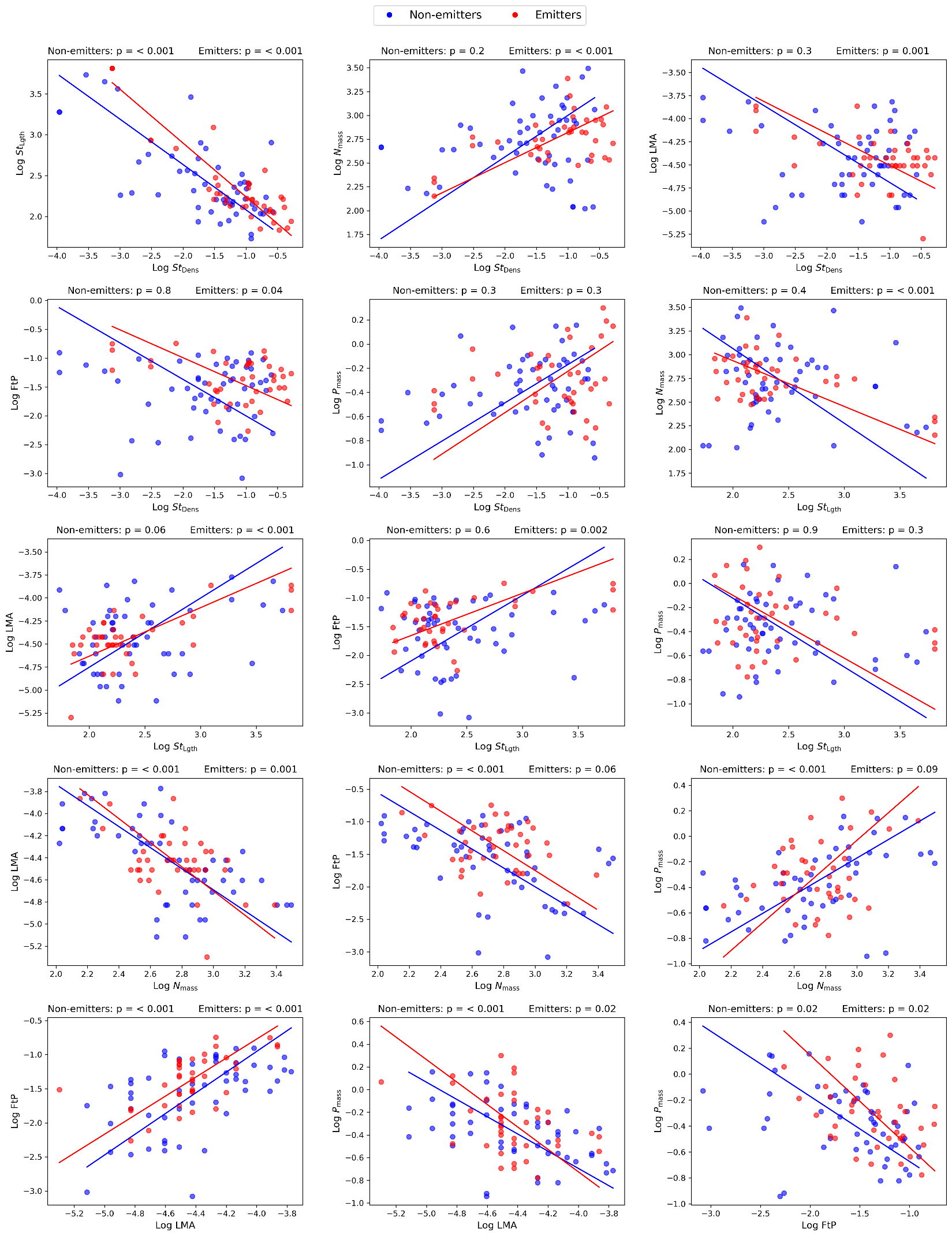
**

**Figure S9.** Results of log-scaled pairwise standard major axis regressions (SMA) for 91 trees from 31 species of angiosperm in a central Amazon forest performed between stomatal density (*St*_Dens_) and leaf and stem traits which showed positive (wood density of the stem, *WD*_st_; force to punch, FtP) and negative correlations with *St*_Dens_ (stomatal conductance, *g*_s_; net photosynthesis per dry mass, *A*_mass_; proportion of sapwood area per basal area, SABA) in the second principal component extracted from the principal component analysis (PCA).

**
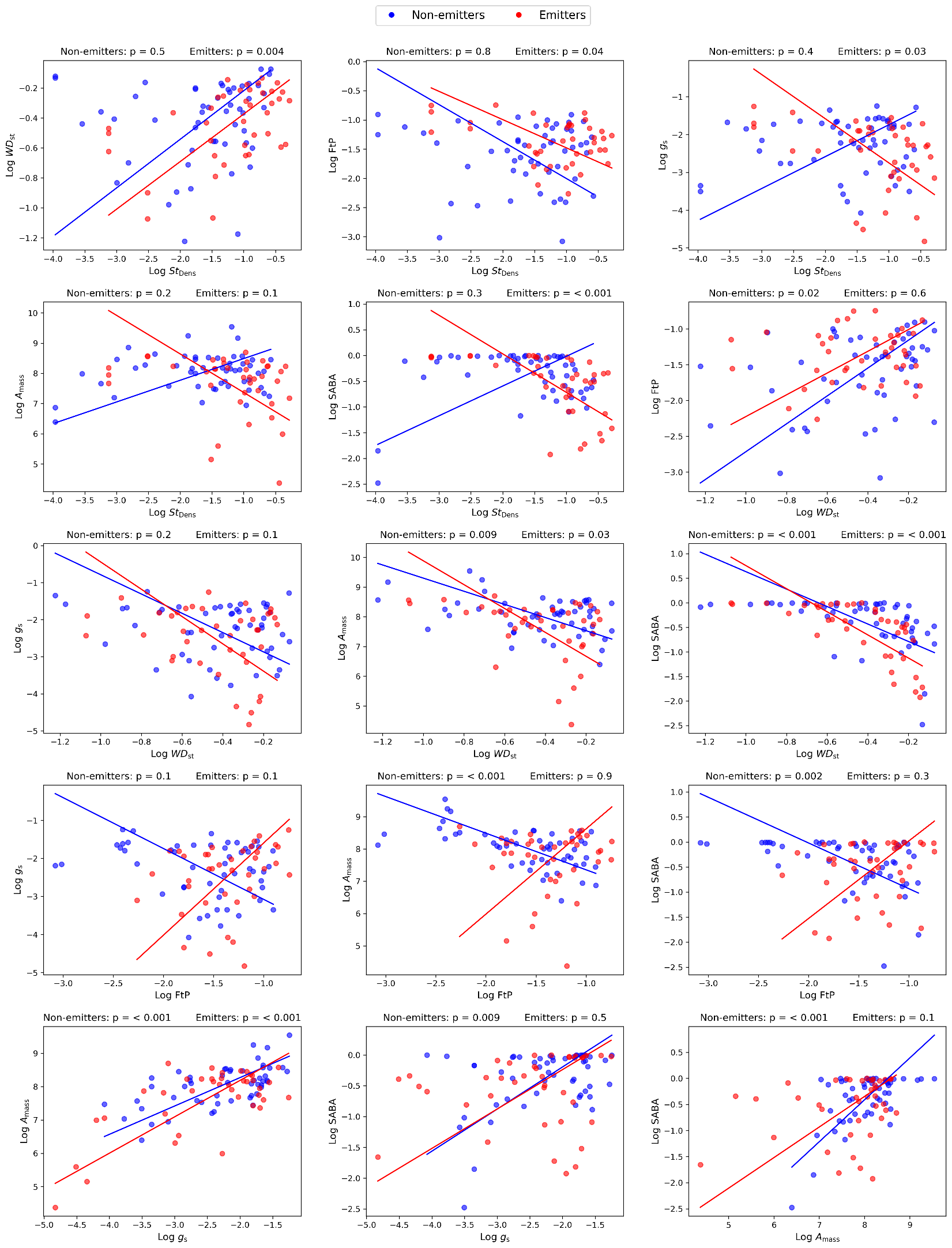
**

**2. Supplementary Tables**

**Table S1.** Air temperature (Air temp, °C), relative humidity (RH, %), photosynthetic active radiation (PAR, µmol m^-2^ s^-1^), and precipitation (mm) for the periods of data collection (campaign days). Values within brackets represent one standard deviation of the mean. Data was obtained from the weather station located at the micrometeorological tower (*Terra firme* forest).

| **Year** | **Month** | **Campaign days** | **Season** | **Air temp °C** | **RH**  **%** | **PAR**  **µmol m^-2^ s^-1^** | **Precipitation mm** |
| --- | --- | --- | --- | --- | --- | --- | --- |
| 2018 | November | 20 - 30 | dry-to-wet transition | 25.9 (0.94) | 94.89 (3.58) | 636.10 (225.79) | 81.8 |
| 2018 | December | 1 - 20 | dry-to-wet transition | 25.06 (0.70) | 94.60 (2.97) | 737.02 (170.44) | 192.5 |
| 2019 | April | 26 - 30 | wet | 25.80 (0.57) | 95.22 (60.70) | 739.14 (131.11) | 28.2 |
| 2019 | May | 1 - 5 | wet | 25.96 (0.65) | 92.41 (5.39) | 754.51 (128.95) | 23.6 |

**Table S2.** Composition and concentration (ppbv) of standard gases used for quantification and identification of isoprene and emitted monoterpenes.

| **Compound** | **Concentration (ppbv)** |
| --- | --- |
| Isoprene | 500.7 ±5% |
| α-Pinene | 477.7 ±5% |
| Camphene | 474.6 ±5% |
| β-Pinene | 500.6 ±5% |
| Myrcene | 353.1 ±5% |
| β-Phellandrene | 380.6 ±5% |
| γ-Terpinene | 522.2 ±5% |
| Limonene | 488.7 ±5% |
| *p*-Cymene | 463.7 ±5% |

**Table S3**. Number of trees measured per species (trees), and number of trees that showed detected emission capacity (*E*_c_) of isoprene, monoterpenes, α-pinene, α-terpinene, β-myrcene, β-phellandrene, β-pinene, camphene and p-cymene per species.

| **Species** | **trees** | **isoprene** | **monoterpenes** | **α-pinene** | **α-terpinene** | **β-myrcene** | **β-phellandrene** | **β-pinene** | **camphene** | **p-cymene** |
| --- | --- | --- | --- | --- | --- | --- | --- | --- | --- | --- |
| *Aldina heterophylla* | 3 | 3 | 3 | 0 | 3 | 0 | 0 | 0 | 0 | 0 |
| *Aspidosperma carapanauba* | 2 | 0 | 2 | 0 | 2 | 0 | 1 | 1 | 0 | 0 |
| *Bocageopsis pleiosperma* | 3 | 0 | 1 | 1 | 0 | 0 | 0 | 0 | 0 | 0 |
| *Chrysophyllum sanguinolentum* | 3 | 0 | 1 | 0 | 1 | 0 | 0 | 0 | 0 | 0 |
| *Corythophora rimosa* | 3 | 3 | 2 | 0 | 1 | 0 | 0 | 0 | 1 | 0 |
| *Croton matourensis* | 3 | 0 | 1 | 1 | 1 | 0 | 0 | 0 | 0 | 0 |
| *Ecclinusa guianensis* | 2 | 0 | 2 | 0 | 2 | 0 | 0 | 1 | 0 | 0 |
| *Eschweilera coriacea* | 5 | 4 | 0 | 0 | 0 | 0 | 0 | 0 | 0 | 0 |
| *Eschweilera grandiflora* | 5 | 3 | 1 | 0 | 0 | 0 | 0 | 0 | 0 | 1 |
| *Geissospermum sericeum* | 3 | 0 | 1 | 0 | 1 | 0 | 0 | 0 | 0 | 0 |
| *Inga alba* | 2 | 2 | 2 | 0 | 2 | 0 | 0 | 0 | 0 | 0 |
| *Macoubea sprucei* | 3 | 0 | 3 | 0 | 3 | 0 | 0 | 2 | 0 | 0 |
| *Macrolobium duckeanum* | 3 | 0 | 3 | 0 | 3 | 0 | 0 | 1 | 0 | 0 |
| *Manilkara bidentata* | 3 | 0 | 1 | 0 | 1 | 0 | 0 | 0 | 0 | 0 |
| *Minquartia guianensis* | 3 | 3 | 0 | 0 | 0 | 0 | 0 | 0 | 0 | 0 |
| *Mouriri duckeana* | 2 | 0 | 1 | 0 | 0 | 0 | 0 | 0 | 0 | 1 |
| *Naucleopsis caloneura* | 3 | 3 | 1 | 0 | 1 | 0 | 0 | 0 | 0 | 0 |
| *Pachira faroensis* | 3 | 3 | 2 | 0 | 0 | 0 | 0 | 2 | 0 | 0 |
| *Pagamea coriacea* | 3 | 0 | 1 | 0 | 0 | 0 | 1 | 1 | 0 | 0 |
| *Parkia igneiflora* | 3 | 0 | 2 | 0 | 2 | 0 | 0 | 1 | 0 | 0 |
| *Pourouma minor* | 2 | 2 | 1 | 0 | 0 | 0 | 0 | 1 | 0 | 0 |
| *Pouteria caimito* | 3 | 0 | 0 | 0 | 0 | 0 | 0 | 0 | 0 | 0 |
| *Pradosia schomburgkiana* | 2 | 0 | 2 | 0 | 2 | 0 | 1 | 1 | 0 | 0 |
| *Protium grandifolium* | 4 | 4 | 2 | 0 | 2 | 0 | 0 | 0 | 0 | 0 |
| *Protium hebetatum* | 5 | 5 | 5 | 0 | 0 | 0 | 5 | 0 | 0 | 0 |
| *Rinorea guianensis* | 2 | 0 | 2 | 0 | 2 | 0 | 0 | 2 | 0 | 0 |
| *Scleronema micranthum* | 3 | 0 | 1 | 0 | 1 | 0 | 1 | 1 | 1 | 0 |
| *Simarouba amara* | 3 | 1 | 2 | 0 | 2 | 0 | 0 | 1 | 1 | 0 |
| *Swartzia reticulata* | 2 | 2 | 2 | 0 | 2 | 1 | 1 | 1 | 0 | 0 |
| *Theobroma sylvestre* | 3 | 1 | 2 | 0 | 0 | 0 | 2 | 0 | 0 | 0 |
| *Trichilia schomburgkiana* | 2 | 0 | 0 | 0 | 0 | 0 | 0 | 0 | 0 | 0 |
| Total | 91 | 39 | 49 | 2 | 34 | 1 | 12 | 16 | 3 | 2 |
